# Deletion of HP1γ in cardiac myocytes affects H4K20me3 levels but does not impact cardiac growth

**DOI:** 10.1186/s13072-018-0187-z

**Published:** 2018-04-17

**Authors:** Kyohei Oyama, Danny El-Nachef, Chen Fang, Hidemi Kajimoto, Jeremy P. Brown, Prim B. Singh, W. Robb MacLellan

**Affiliations:** 1 Division of Cardiology, Department of Medicine, Center for Cardiovascular Biology and Institute for Stem Cell and Regenerative Medicine, University of Washington, 1959 NE Pacific St, Box 356422, Seattle, WA 98195-6422 USA; 2 Fächerverbund Anatomie, Institut für Zell-und Neurobiologie, Charite-Universitätsmedizin, 10117 Berlin, Germany; 3 Department of Biomedical Sciences, Nazarbayev University School of Medicine, Astana, Kazakhstan 010000; 4 Department of Natural Sciences, Laboratory of epigenetics, Novosibirsk State University, Pirogova str. 1, Novosibirsk, 630090 Russian Federation

**Keywords:** HP1γ, Conditional knockout, Cardiac myocytes, Cell cycle, Gene expression, H4K20me3, H3K9me3

## Abstract

**Background:**

Heterochromatin, which is formed when tri-methyl lysine 9 of histone H3 (H3K9me3) is bound by heterochromatin 1 proteins (HP1s), plays an important role in differentiation and senescence by silencing cell cycle genes. Cardiac myocytes (CMs) accumulate heterochromatin during differentiation and demethylation of H3K9me3 inhibits cell cycle gene silencing and cell cycle exit in CMs; however, it is unclear if this process is mediated by HP1s. In this study, we created a conditional CM-specific HP1 gamma (HP1γ) knockout (KO) mouse model and tested whether HP1γ is required for cell cycle gene silencing and cardiac growth.

**Results:**

HP1γ KO mice were generated by crossing HP1γ floxed mice (fl) with mice expressing Cre recombinase driven by the Nkx2.5 (cardiac progenitor gene) promoter (Cre). We confirmed that deletion of critical exons of HP1γ led to undetectable levels of HP1γ protein in HP1γ KO (Cre;fl/fl) CMs. Analysis of cardiac size and function by echo revealed no significant differences between HP1γ KO and control (WT, Cre, fl/fl) mice. No significant difference in expression of cell cycle genes or cardiac-specific genes was observed. Global transcriptome analysis demonstrated a very moderate effect of HP1γ deletion on global gene expression, with only 51 genes differentially expressed in HP1γ KO CMs. We found that HP1β protein, but not HP1α, was significantly upregulated and that subnuclear localization of HP1β to perinuclear heterochromatin was increased in HP1γ KO CMs. Although HP1γ KO had no effect on H3K9me3 levels, we found a significant reduction in another major heterochromatin mark, tri-methylated lysine 20 of histone H4 (H4K20me3).

**Conclusions:**

These data indicate that HP1γ is dispensable for cell cycle exit and normal cardiac growth but has a significant role in maintaining H4K20me3 and regulating a limited number of genes in CMs.

**Electronic supplementary material:**

The online version of this article (10.1186/s13072-018-0187-z) contains supplementary material, which is available to authorized users.

## Background

Adult cardiac myocytes (ACMs) are terminally differentiated cells that express a unique cell-specific transcriptional profile. During terminal differentiation of CMs, cell cycle genes are irreversibly silenced [1, 2] by a mechanism which we have hypothesized is similar to what occurs in cellular senescence [3, 4]. Induction of senescence is thought to involve the sequestration of cell cycle genes within senescence-associated heterochromatin foci (SAHF), which are enriched with heterochromatic markers such as tri-methyl lysine 9 of histone H3 (H3K9me3), tri-methyl lysine 20 of histone H4 (H4K20me3), macroH2A and heterochromatin protein 1 (HP1) [5, 6]. For example, E2F target genes that are required for proliferation localize to SAHF [7] and are enriched with heterochromatic marks [5]. Because heterochromatin acts as a physical barrier, cell cycle genes are thought to no longer be accessible to transcriptional machinery that drive expression and thereby lead to exit of the cell cycle.

Heterochromatin assembles not only into large domains of constitutive heterochromatin but can also regulate gene activity on a gene-by-gene basis. For example, the master cell cycle regulator retinoblastoma protein (RB) [8, 9] recruits key players of heterochromatin formation such as Suv39h1 H3K9 histone metyltransferase (HMT), HP1s and histone deacetylases [4, 5, 10–12] to E2F target genes and assemble localized heterochromatin complexes. Indeed, loss of RB impaired SAHF formation [5]. RB is one of member of gene family encoding three structurally and functionally similar proteins, RB, p107 and p130. In ACMs, RB and p130 are expressed [1, 4]. We previously knocked out both RB and p130 in ACMs and found that RB/p130 double KO (RB/p130-DKO) ACMs failed to silence cell cycle genes and reentered cell cycle [4]. In addition, cardiac-specific H3K9me3 depletion prevented both cell cycle exit and terminal differentiation [13]. These results indicate that heterochromatin formation through H3K9me3 could mediate cell cycle exit in CM terminal differentiation.

**Fig. 1:**
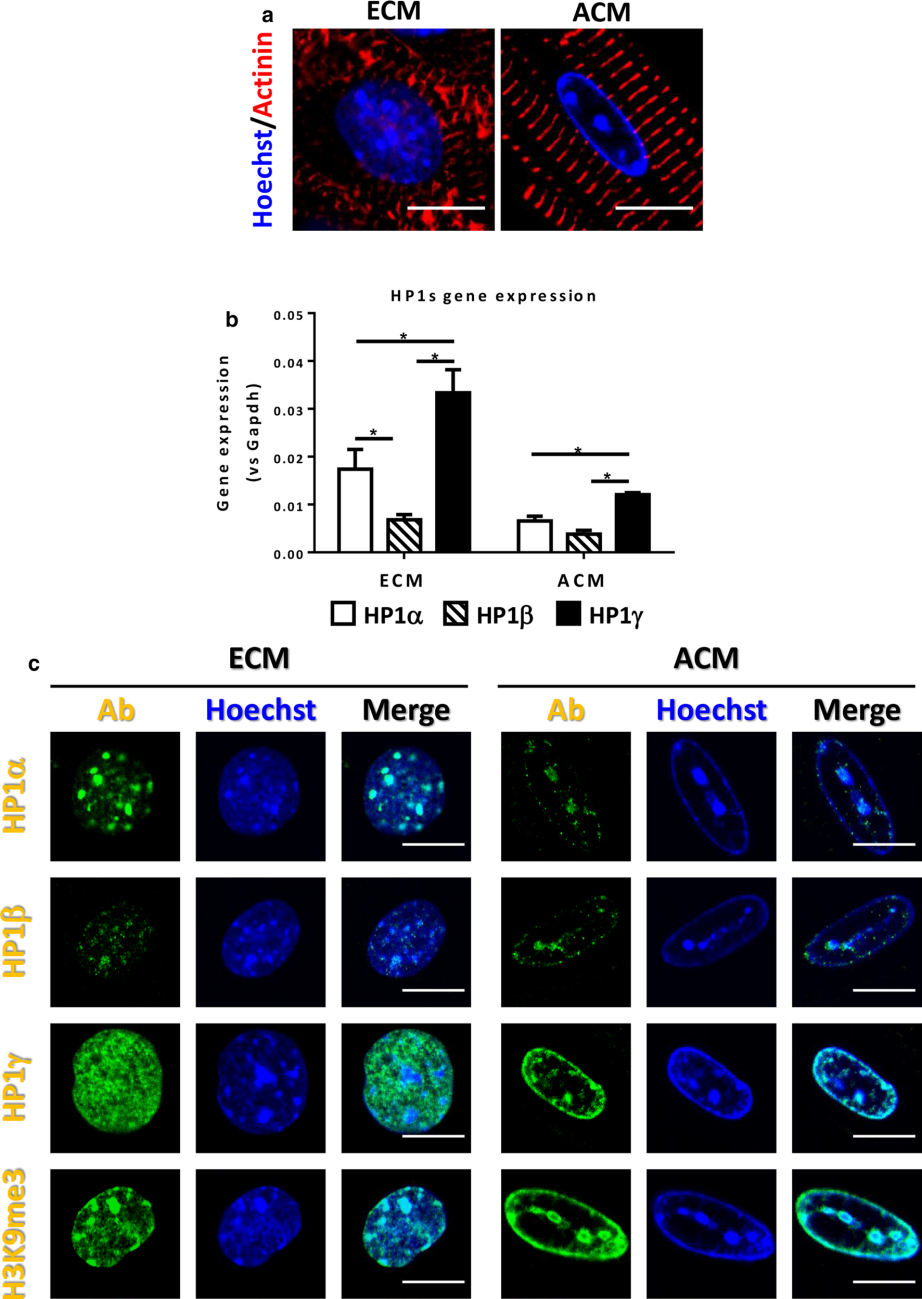
Characterization of HP1s in cardiac development. **a** Heterochromatin accumulation during cardiac development. Heterochromatin was visualized by Hoechst staining (Blue) and CM-specific maker α-actinin was immunostained (Red). Scale bar indicates 10 μm. **b** Comparison of HP1s gene expression. Total RNA was isolated from purified ECM and ACM. Gene expression levels were measured by using isoform-specific primer sets for qPCR following reverse transcription. Expression levels were compared to GAPDH gene expression. *p < 0.05. **c** Localization of HP1s in CMs. ECMs and ACMs were isolated and HP1s and H3K9me3 were stained using specific antibodies (Green). DNA was stained with Hoechst to visualize heterochromatin foci (Blue). Scale bar indicates 10 μm

HP1 is the first non-histone heterochromatin factor identified as a dosage-dependent modifier of position-effect variegation in *Drosophila* [14, 15]. There are three isoforms in mammals (HP1α, HP1β and HP1γ) and all isoform share two conserved domains, the chromo domain (CD) and the chromo shadow domain (CSD). HP1s directly bind to H3K9me2/3 through the CD and simultaneously recruit Suv39h1 via CSD [16–21], which allows HP1s to propagate and establish heterochromatin [22]. HP1γ is the most highly expressed among the three isoforms and is the only isoform showing co-localization with H3K9me3 in ACMs (Fig. 1 and Additional file 1: Fig. S1), but its physiological role and importance in vivo are unknown.

In this study, we tested the hypothesis that HP1γ is required for the cell cycle gene silencing in CMs. Accordingly, we generated a novel conditional mouse model where HP1γ was specifically deleted in CMs. Surprisingly, loss of HP1γ had minimal effect on cardiac gene expression and differentiation.

## Methods

### Creation of Nkx2.5-Cre;HP1γfl/fl

The HP1γfl mouse was generated from the HP1γ^hypo/hypo^ [23] by excision of the FRT-NeO-FRT cassette after crossing to a FLPe mouse [24]. The resulting HP1γfl mouse was crossed to the Nkx2.5-Cre mouse [25] to achieve cardiac-specific HP1γ KO. Litter mates were used as controls for this study. Mice were housed in a temperature-controlled environment with 12 h light/dark cycles where they received food and water ad libitum. All protocols concerning the use of animals were approved by the Institutional Animal Care and Use Committees at University of Washington.

### Cardiac myocyte isolation

Embryonic CMs were isolated by enzymatic digestion. Briefly, E15-timed pregnant female mice were euthanized and embryonic hearts were extracted from embryos and pooled into ice-cold Ads buffer (116 mM NaCl, 20 mM HEPES, 11 mM NaH_2_PO_4_, 5.5 mM Glucose, 5.4 mM KCl, 0.83 mM MgSO_4_, pH 7.4) supplemented with 5 U/ml heparin. Hearts were washed and atriums were removed in Ads buffer with heparin. The ventricles were minced and digested with 5 mg/ml collagenase Type II (Worthington, 4176) and 1 mg/ml pancreatin (Sigma, P3292) prepared at 37 °C for 20 min. Cardiac myocytes were purified using a Percoll gradient, resulting in ~ 90% pure CMs.

Adult CMs were isolated by using Langendorff perfusion digestion. Briefly, adult hearts were washed by perfusing with Ca^2+^ free Tyrode’s buffer (126 mM NaCl, 5.4 mM KCl, 0.33 mM NaH_2_PO_4_, 1 mM MgCl_2_, 10 mM HEPES, 10 mM Glu, 20 mM taurine, pH 7.4) supplemented with 25 μM blebbistatin (Toronto Research Chemicals Inc. and BOC Sciences) for 1–2 min and then enzymatically digested at 37C by perfusing with 0.69 U/ml Liberase TH (Roche) prepared with blebbistatin supplemented-Ca^2+^ free Tyrode’s buffer for 8–12 min. Hearts were mechanically dissociated in ice-cold KB solution (20 mM KCl, 10 mM KH_2_PO_4_, 70 mM potassium glutamate, 1 mM MgCl_2_, 25 mM Glucose, 20 mM Taurine, 0.5 mM EGTA, 10 mM HEPES, 0.1% albumin, pH 7.40). CM suspensions were passed through a 100–200 μm cell strainer to remove tissue debris and then purified by low-speed centrifugation (50×*g* for 1 min) 3 times, resulting in ~ 90% pure ACMs.

### Protein analysis

#### Nuclear extracts were prepared from isolated CMs

Purified ACMs were resuspended in NP40 lysis buffer (0.5% NP40, 25 mM KCl, 3 mM MgCl_2_, 10 mM Tris–HCl, pH 8.0) and homogenized until nuclei were released from the cytoskeleton. Extraction buffer was supplemented with 1 mM Na_3_VO, 1 mM NaF, 1 mM phenylmethylsulfonyl fluoride and 1× Protease Inhibitor Cocktail (Millipore 539134). The homogenate was centrifuged with 50×*g* for 1 min at 4 °C and supernatant (enriched nuclear) was corrected. The enriched nuclear was pelleted with 800×*g* for 10 min at 4 °C. Nuclear pellets were resuspended in SDS/βME nuclear buffer (1% SDS, 25 mM 2-mercaptoethanol, 137 mM NaCl, 0.5% NP40, 25 mM KCl, 3 mM MgCl2, 10 mM Tris–HCl, pH 8.0) and sonicated with probe sonicator (Qsonica, CL-18) with 25% power for 10 s 2 time (30 s interval). Nuclear extracts were cleared by centrifugation at 20,000×*g* for 10 min at 15 °C. DNA concentration was measured using Quant-iT™ PicoGreen^®^ dsDNA Reagent (Life technology) and used to normalize samples. Nuclear extract (50–250 ng DNA) were separated by SDS–PAGE, transferred to polyvinylidene fluoride membrane and probed using specific primary antibodies and appropriate HRP-conjugated secondary antibody for ECL detection. Antibodies used are listed in Additional file 3.

### Immunofluorescence staining

Isolated CMs were fixed with cold methanol. Heart tissues were fixed with 4% PFA and paraffin- embedded. Fixed CMs and paraffin-embedded tissue sections were immunostained following standard protocols. Briefly, fixed CMs and deparaffinized/rehydrated tissues were blocked with 1% bovine serum albumin (BSA)/phosphate buffered saline (PBS). Primary antibodies were diluted in 1% BSA/PBS and incubated with blocked CMs and tissues for overnight at 4 °C. Appropriate secondary antibodies conjugated with Alexa Fluor^®^ Dyes (Life Technologies) were diluted in 1% BSA/PBS and incubated with primary antibodies-probed CMs and tissues for 1 h at room temperature. Nuclei were counter stained with Hoechst (Life Technologies). Where indicated, heart tissues were stained with wheat germ agglutinin (WGA) conjugated with Oregon Green™ 488 (Thermo) to visualize cell border. Fluorescence images were acquired using a confocal microscope (Nikon A1R).

### RNA analysis

Total RNA was extracted from isolated CMs. All total RNA were treated with DNase I following manufacture’s protocol to avoid genomic DNA contamination. Embryonic CMs total RNA were extracted using RNeasy Micro Kit (Qiagen). Adult CMs total RNA was pre-cleared using TRIzol™ Reagent (Invitrogen) and then purified using RNeasy Micro Kit (Qiagen). 1 μg of total RNA was converted to cDNA using Transcriptor First Strand cDNA Synthesis Kit (Roche). cDNA corresponding to 5 ng of total RNA was used for quantitative PCR. Quantitative PCR was performed using SYBR™ Select Master Mix (Life Technologies). Primer sequences used in this study are listed in Additional files 2 and 3. PCR specificity was confirmed by amplicon dissociation curve and by running amplicon on agarose gel. Standard curves were created for each assay. Target gene expression was normalized against internal control S26 or GAPDH and presented as relative expression value against control sample as described in figure legend after confirming expression was similar between the genotypes or operations we used in this study.

### Transverse aortic constriction (TAC) surgery

10–12 week old mice were subjected to TAC surgery using a 27-gauge needle as previously described [26, 27] and hearts were harvested 1 week after surgery.

### RNA-seq

8 week baseline RNA-seq: Total RNA were extracted from 3 HP1γ KO CMs and 3 controls CMs (WT, Cre and fl/fl, one of each) at 8 week old. Library construction and sequencing was performed using commercial service (Omega Bioservices). Briefly, sequenced reads were mapped on the mouse genome (mm10) assembly using TopHat [28]. Transcripts were assembled and fragments per kilobase of transcript per million (FPKM) was calculated by Cufflinks [29]. Differential gene expression was calculated using Cuffdiff [29]. We defined differentially expressed gene as false discovery rate (FDR) < 0.05 and fold change ≥ 2.

TAC-Operated CMs RNA-seq: Three independent samples per group were used: Sham-fl/fl, Sham-Cre;fl/fl, TAC-fl/fl and TAC-Cre;fl/fl. Library construction and sequencing was performed by the Genomics Core Service at Fred Hutchinson Cancer Research Center (Seattle, WA). Sequenced reads were mapped on mouse mm10 using TopHat, counts for each gene were generated using HTSeq [30], and normalization and comparison of gene expression difference were conducted by edgeR [31, 32]. Genes with FDR < 0.05 and fold change ≥ 2 are defined as significantly differentially expressed.

## Results

### HP1γ is the major HP1 isoform in ACM

To determine if heterochromatin accumulates in CMs during terminal differentiation (Fig. 1a) CMs from E15 embryo and 10-week adult mouse hearts were stained with Hoechst dye, which stains AT-rich DNA that is concentrated within heterochromatin [33]. E15 embryonic CMs (ECMs) showed a diffuse staining pattern with multiple small heterochromatin foci. In contrast, ACMs showed accumulation of heterochromatin at the nuclear periphery with a few large internal heterochromatin foci.

To explore the role of HP1s in heterochromatin accumulation in CMs further we characterized expression of HP1s during cardiac development. We performed RT-qPCR using purified CMs to compare expression of each HP1 isoform (Fig. 1b). All three HP1 isoforms are expressed in both ECM and ACM. Of these isoforms, HP1γ demonstrated the highest gene expression in both ECM and ACM (1.9-fold vs HP1α; *p* = 0.0016 and 4.8-fold vs HP1β; *p* = 0.0002 in ECM, 1.8-fold vs HP1α; *p* = 0.0165 and 3.1-fold vs HP1β; *p* = 0.0013 in ACM).

Next, we compared the nuclear localization pattern of HP1s (Fig. 1c). In ECMs, HP1α and HP1β are localized with heterochromatin foci (Hoechst), but HP1γ showed diffuse staining which is euchromatic pattern. In contrast, in ACMs, HP1α and HP1β staining was speckled and no longer confined to heterochromatin foci, while HP1γ staining pattern overlapped with heterochromatin, which is similar to H3K9me3 staining. Co-staining HP1s with H3K9me3 further confirmed that HP1γ is localized with H3K9me3 coincident with heterochromatin foci in ACMs (Additional file 1: Fig. S1). These data demonstrate that HP1γ is the major isoform of HP1s that co-localizes with H3K9me3 in ACMs, suggesting it may play a role in heterochromatin organization during terminal differentiation of CMs.

### Creation of cardiac myocyte-specific HP1γ KO mice

**Fig. 2:**
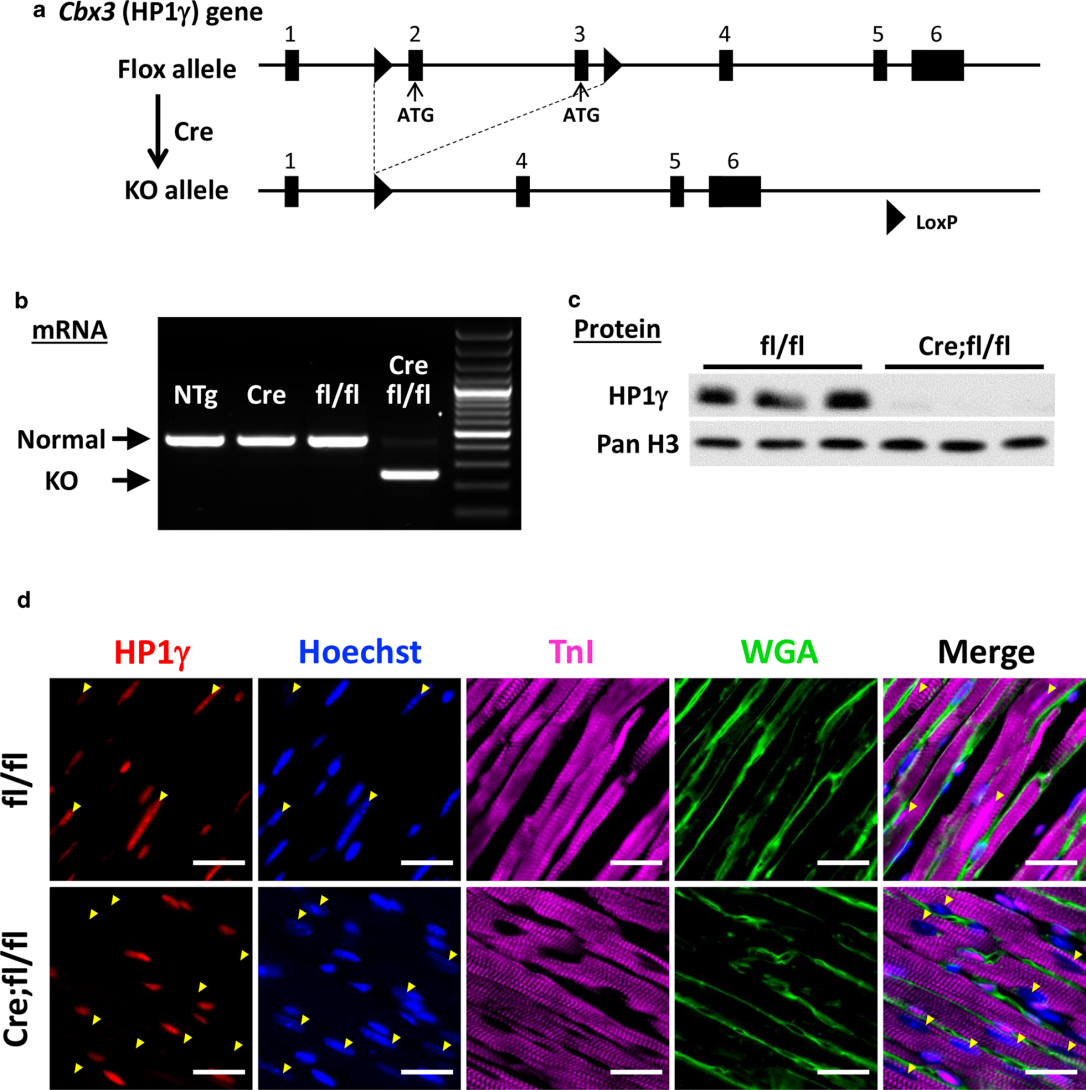
Generation of a cardiac-specific HP1γ KO mouse. **a** Scheme of conditional HP1γ KO strategy. **b** Confirmation of critical exons deletion. Total RNA was extracted from purified 8-week ACMs and RT-PCR was performed. Longer amplicon indicates normal mRNA and shorter amplicon indicates KO mRNA. Molecular weight maker shows 100 bp ladder. **c** Confirmation of HP1γ protein loss. Western blotting was performed on nuclear extracts from purified CMs. **d** Specificity of HP1γ KO in heart tissue. Heart tissues were staining using HP1γ specific antibody (Red). Hoechst staining is in blue, WGA staining is in green and cardiac TnI staining is in magenta. Yellow arrowheads indicate CM nuclear. Scale bar indicates 25 μm

Germline HP1γ-deficient mice (hypomorphic allele and gene trap KO) [23, 34, 35] rarely survive to adulthood [23], suggesting that HP1γ has an important non-redundant function with respect to the other HP1 family members. To determine its role in tissue-specific development, we created a conditional cardiac HP1γ KO mouse (Cre;fl/fl) driven by Nkx2.5-Cre (Cre). Nkx2.5 is a cardiac progenitor specific transcription factor and is first expressed in CMs at E7.5 [25]. HP1γ floxed mice (HP1γfl) were engineered with loxP sites flanking exon 2 and exon 3, which includes the start codon and an alternative in frame ATG so that no translation of HP1γ occurs (Fig. 2a) [23]. Deletion of exons 2–3 in CMs was confirmed by RT-PCR (Additional file 4: Fig. S2A). Generation of HP1γ KO CMs demonstrated that the recombined transcript and normal RNA was almost undetectable (Fig. 2b), indicating highly efficient deletion. In contrast all control CMs (WT, Cre and homozygous HP1γfl (fl/fl)) showed wild-type transcripts. Consistent with deletion of exon 2–3, HP1γ protein was undetectable in KO CMs (Fig. 2c). As shown in Fig. 2d, HP1γ is expressed ubiquitously in both CM (yellow arrow head) and non-CM in control (fl/fl) hearts. In contrast, HP1γ expression is absent in CMs of HP1γ KO hearts. Unexpectedly, although the Nkx2.5 has been reported to be cardiac specific, we observed substantial recombination of HP1γ in liver (Additional file 4: Fig. S2B) [25].

### Loss of HP1γ causes reduction in H4K20me3 and upregulation of HP1β

**Fig. 3:**
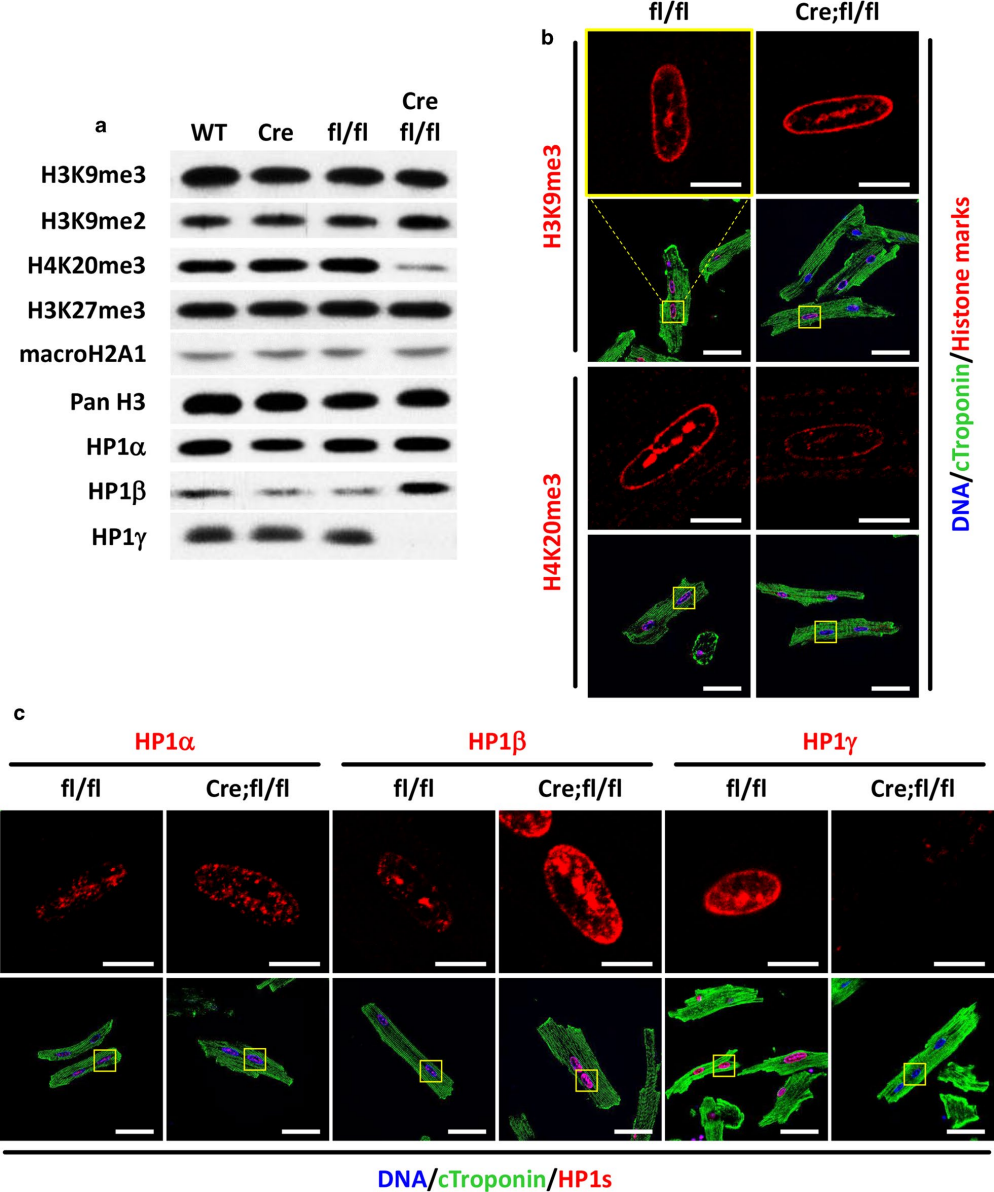
Effect of HP1γ KO on heterochromatin formation. **a** Expression level of heterochromatin markers. Nuclear extract was prepared from purified CM at 8 week and 100–250 ng DNA was loaded per lane. Representative pictures of 5 biological independent experiments are shown here. **b**, **c** Localization of heterochromatic histone marks and HP1s. 8-week CMs from fl/fl control and KO (Cre;fl/fl) mice were isolated and stained with specific antibodies. Single channel images (zoomed in) of histone marks and HP1s are shown in upper panel in red. Cardiac troponin is in green (lower panel) and DNA is in blue (lower panel). Individual channel images are available in Additional file 6: Fig. S4. Scale bar in upper panel and lower panel indicate 10 and 50 μm, respectively

Since HP1γ recruits H3K9 HMTs, G9a and Suv39h1 that are responsible for H3K9me2 and H3K9me3, respectively, which allows extension of H3K9 methylation to adjacent histones [19, 21, 36, 37], we examined if loss of HP1γ effects the methylation status of H3K9. Nuclear lysates were prepared using isolated CMs from HP1γ KO (Cre;fl/fl) and controls (WT, Cre and fl/fl). Western blotting demonstrated that there was no difference in total H3K9me3 or H3K9me2 (Fig. 3a and Additional file 5: Fig. S3). Stable heterochromatin formation requires sequential methylation of H4K20me3 after H3K9me3, which is also dependent on HP1s [38, 39]. We tested if loss of HP1γ effects H4K20me3 status and found that HP1γ deletion resulted in a 75% reduction in global H4K20me3 levels (Fig. 3a) compared to control groups (*p *< 0.015 vs WT, Cre and fl/fl; Additional file 5: Fig. S3). H4K20me3 immunostaining signal was also reduced in HP1γ KO CMs consistent with the immunoblotting; although there was no change in H3K9me3 immunostaining (Fig. 3b and Additional file 6: Fig. S4A). Neither macroH2A, a heterochromatin component nor H3K27me3, a marker of facultative heterochromatin, were affected (Fig. 3a). We did not detect signs of DNA damage (γH2AX, p21) or senescence (p16) in HP1γ KO CMs (Additional file 7: Fig. S5).

Next, we tested if loss of HP1γ effected the other isoforms of HP1s, HP1α and HP1β. HP1α protein level was unchanged between controls and HP1γ KO (Fig. 3a). However, HP1β protein expression was 2.3 times higher in HP1γ KO CM compared to controls (*p *< 0.01 vs WT, Cre and fl/fl; Additional file 5: Fig. S3). Immunofluorescence staining demonstrated that there is no difference of HP1α localization pattern or signal intensity. In contrast, HP1β was increased and changed in localization to the nuclear periphery (Fig. 3c and Additional file 6: Fig. S4B). Thus, loss of HP1γ affects HP1β expression and localization, but not HP1α.

### HP1γ depletion has no effect on cell cycle gene expression and cardiac growth

**Fig. 4:**
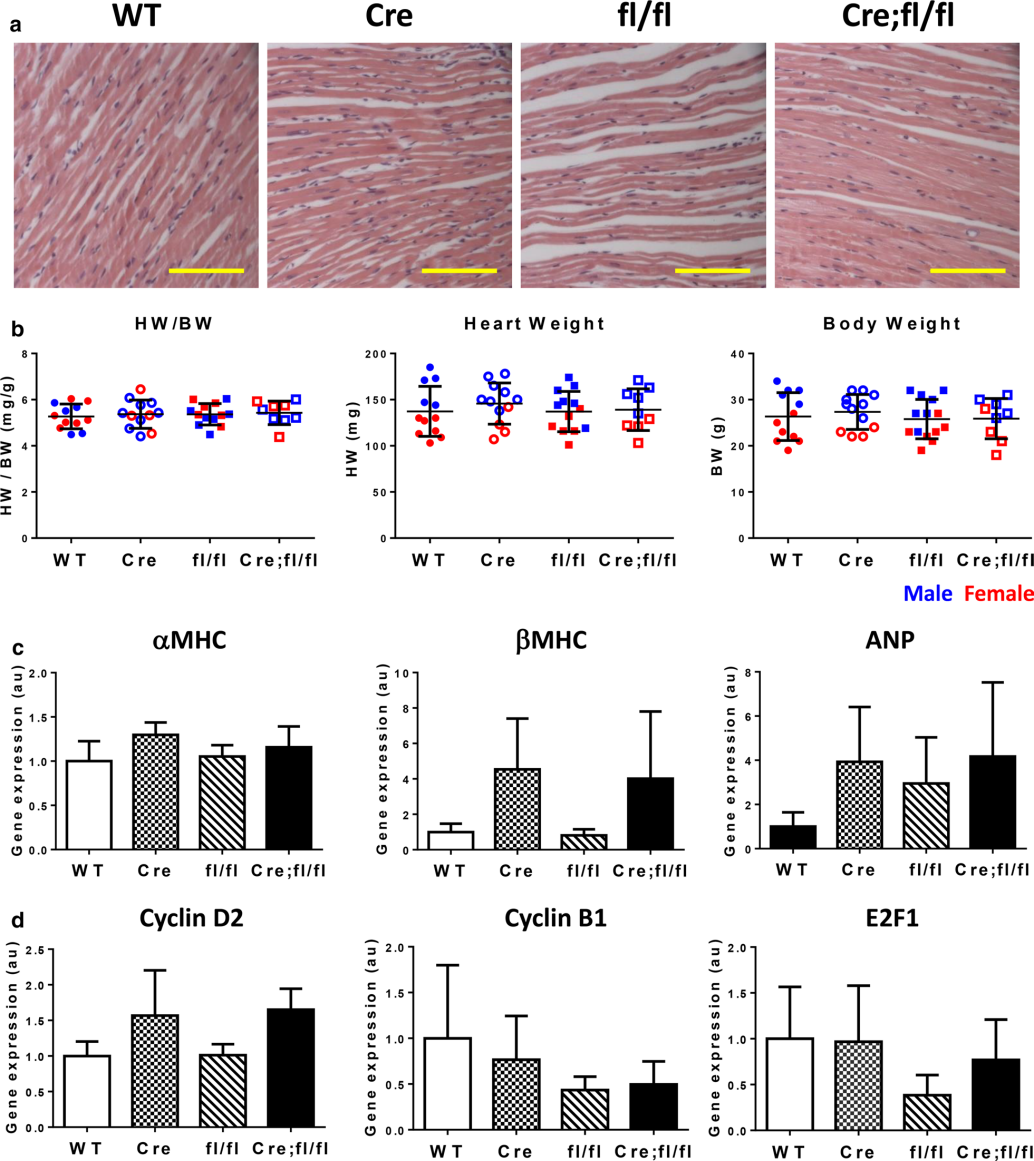
Characterization of role of HP1γ in cardiac growth. **a** Hematoxylin and eosin staining of heart tissue. Scale bar indicates 100 μm. **b** Comparison of heart growth. Heart weight and BW were measured at 8-week of age. Each data point indicates a value from individual animal. Data from male animal is in blue and female is in red. **c**, **d** Quantification of cardiac and cell cycle gene expression. Total RNA was extracted from purified ACM at 8-week. Quantitative RT-PCR was performed using specific primers. Gene expression was normalized against expression of internal control S26 and shown as relative expression value against WT control. S26 expression did not change between genotypes

To examine if KO of HP1γ had an effect on cardiac growth we examined the hearts of 8-week old mice. There were no differences in histology between HP1γ KO hearts and control hearts (Fig. 4a) nor was there a difference in heart weight (HW) normalized to body weight (HW/BW) (Fig. 4b). Normalized HW was 5.3 ± 0.45 mg/g, 5.4 ± 0.46 mg/g, 5.5 ± 0.50 mg/g and 5.4 ± 0.41 mg/g in WT, Cre, fl/fl and Cre;fl/fl, respectively. Cardiac function was measured by echocardiography (Additional file 8: Table S1) and no significant difference in cardiac function was observed between HP1γ KO and controls. Since a regulatory role for HP1γ on fetal cardiac genes (ANP) [40] has been reported, we tested the effect of HP1γ depletion on cardiac gene expression (Fig. 4c). There were no significant differences in expression of either adult cardiac genes (αMHC) or fetal cardiac genes (βMHC and ANP), suggesting little impact on cardiac differentiation. Likewise, there was no significant difference in expression of any cell cycle genes.

### Impact of HP1γ loss on global gene expression

**Fig. 5:**
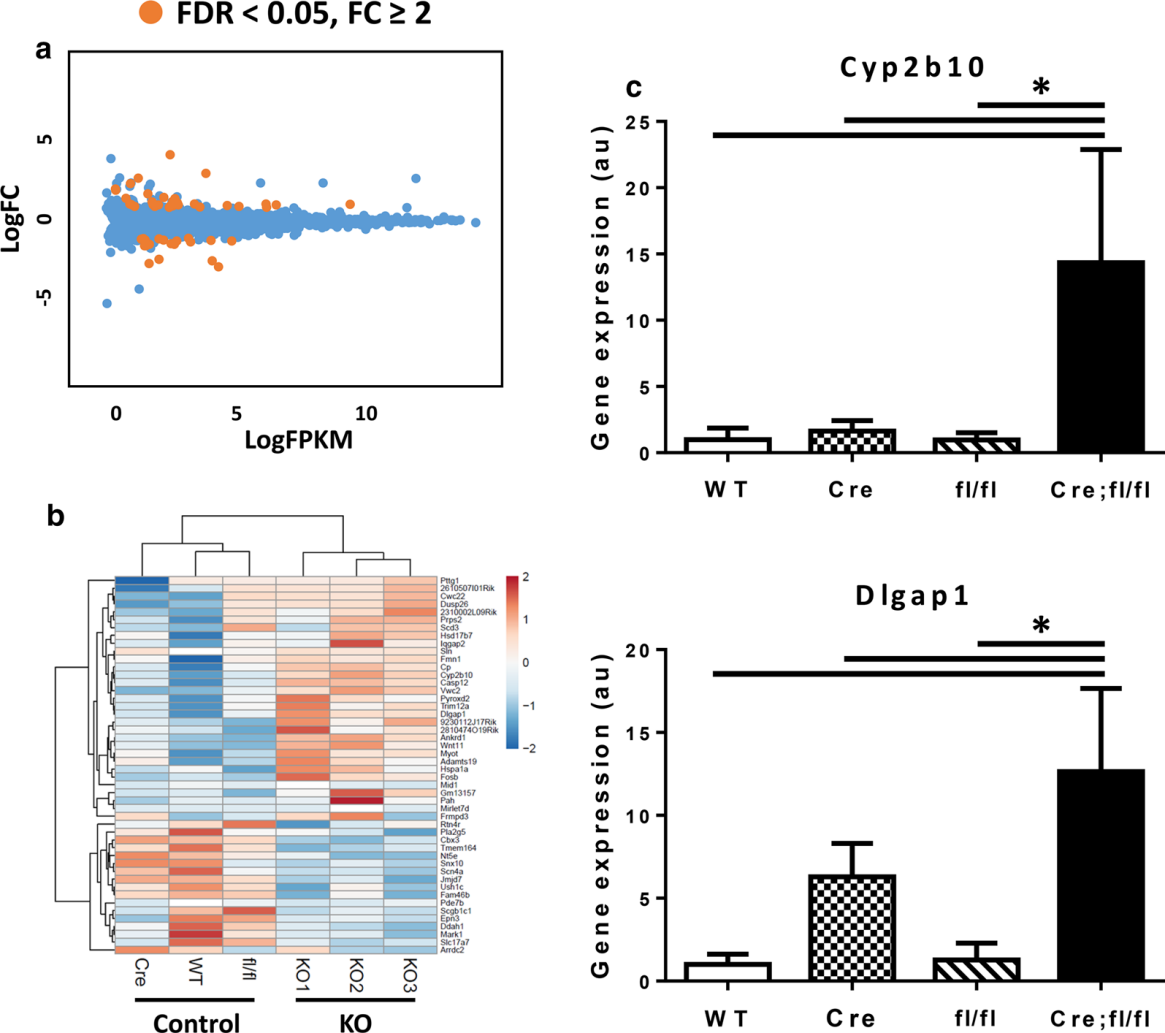
Global gene expression profile in HP1γ KO CMs. **a** Global gene expression changes. Total RNA was isolated from purified CMs at 8-week and RNA-seq was performed. Three biological replicates of HP1γ KO CM was compared to three controls (WT, Cre and fl/fl one of each). Each dot represents averaged expression (log2 FPKM) on *X*-axis and fold change (FC) on *Y*-axis (log2 FC) of RefSeq genes. Orange dots represent differentially expressed genes in HP1γ KO CM compared to control CM (> twofold change with FDR < 0.05). **b** Heat map of differentially expressed genes in HP1γ KO CM. Heat map was created by clustering of differentially expressed gene (FC > 2 and FDR < 0.05). Warmer color and colder color indicate up regulation and down regulation, respectively, in HP1γ KO. **c** Representative differentially expressed genes confirmed by qRT-PCR. *p < 0.05

To assess the effect of HP1γ KO on global gene expression, we performed RNA-seq on HP1γ KO CMs and grouped control CMs (3 genotypes: WT, Cre and fl/fl). Principal component analysis (PCA) [41] showed no clear separation between HP1γ KO and controls, suggesting that no major differences in global gene expression pattern (Additional file 9: Fig. S6). Differential gene expression analysis identified 51 genes with ≥ twofold change and FDR < 0.05 (Fig. 5a). Of these, 32 genes were upregulated and 19 genes were downregulated. Gene ontology enrichment analysis did not identify any biological processes represented by these genes that was affected by loss of HPγ (Additional file 10). These differentially expressed genes were clustered [41] and the resulting heat map demonstrated distinct clustering of HP1γ KO and control, confirming that KO of HP1γ, rather than Cre or fl/fl influenced these changes in gene expression (Fig. 5b). However, most of the differentially expressed genes had relatively small changes in absolute levels and only 11 genes showed ≥ fourfold difference (Fig. 5a and Additional file 10). To confirm these changes, we analyzed levels of Cyp2b10 and Dlgap1 and found they were upregulated 14-fold and 12-fold, respectively (Fig. 5c). These data suggest that loss of HP1γ had a minor impact on global gene expression in CMs.

### A limited effect of HP1γ KO on hypertrophic growth of heart

**Fig. 6:**
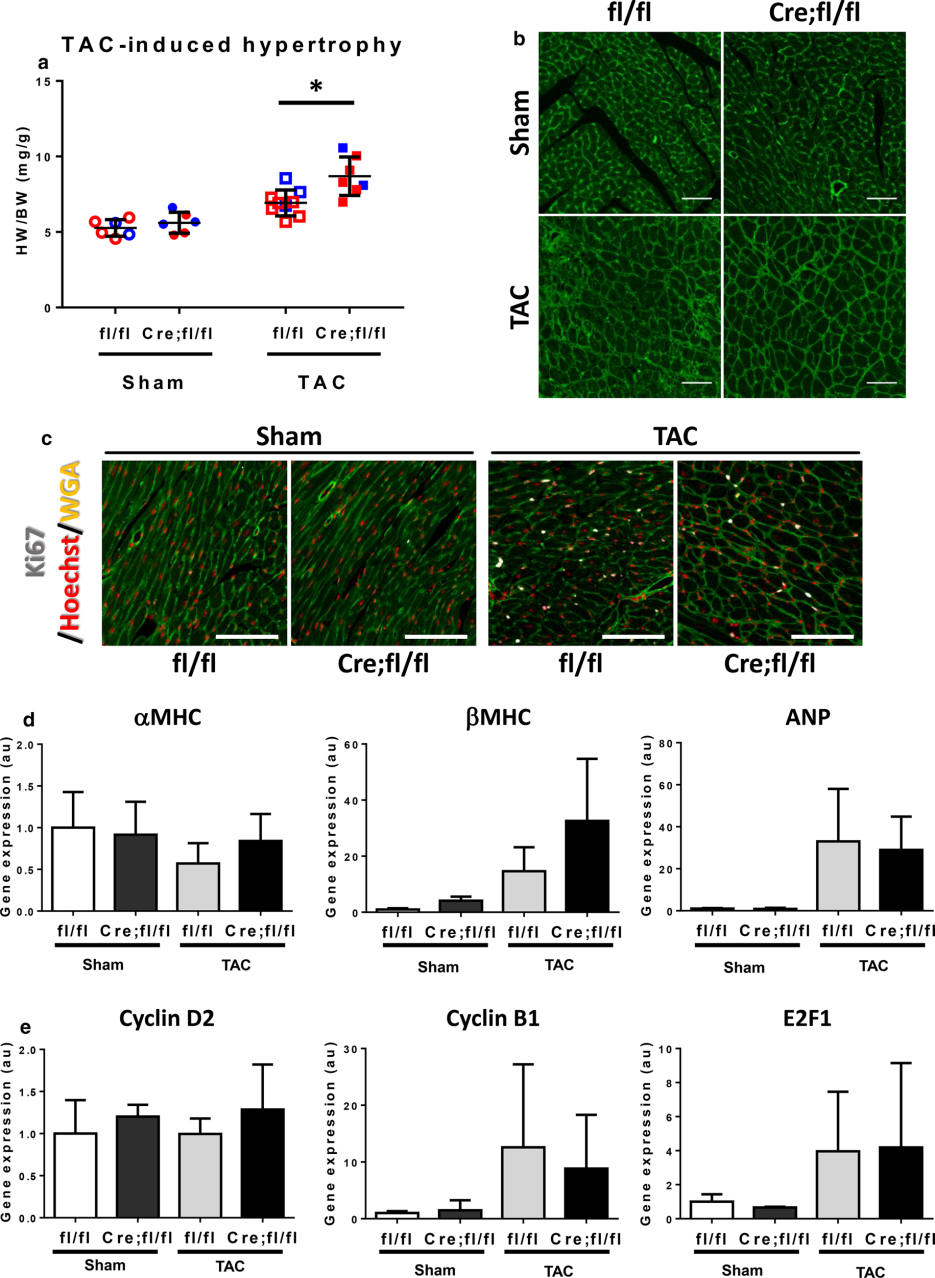
Effect of HP1γ KO on TAC-induced hypertrophy. **a** TAC-induced heart growth. TAC or Sham surgeries were performed at 10–12 weeks and hearts were harvested 1 week after operations. Heart weight were measured and normalized by BW. Each data point indicates a value from individual animals. Data from male are shown in blue and females in red. *p < 0.05. **b** Cross-sectional area after TAC. Paraffin heart sections were stained with WGA. Scale bar indicates 50 μm. **c** Heart tissues were stained with cell cycle marker Ki67 (white), Hoechst (red) and WGA (green). Individual channel images are available in Additional file 12: Fig. S8. Scale bar indicates 100 μm. **d**, **e** Expression of cardiac and cell cycle genes. Total RNA was extracted from isolated CMs 1 week after TAC and quantitative RT-PCR was performed using gene specific primers. Gene expression was normalized against expression of internal control S26 and shown as relative expression value against Sham operated fl/fl. S26 gene expression did not change by neither operation nor genotype

To examine the effect of HP1γ KO on cardiac growth after hemodynamic stress, we performed TAC or Sham surgery on 10–12-week old mice and harvested the hearts seven days later. In all genotypes, TAC increased HW/BW significantly compared to Sham groups and HP1γ KO mice showed slightly higher HW/BW after TAC compared to controls (8.7 ± 1.0 mg/g (TAC-KO) vs 6.9 ± 0.61 mg/g (TAC-control)). We measured cross-sectional area of ACMs to estimate cardiac myocyte size and found CMs in TAC groups were larger than the sham groups but no difference between TAC groups (Fig. 6b). Areas of CMs were 211 ± 26 um^2^, 199 ± 23 um^2^, 289 ± 20 um^2^ and 298 ± 6.5 um^2^ in Sham-control, Sham-HP1γ KO, TAC-control and TAC-HP1γ KO, respectively (Additional file 11: Fig. S7A). There was no significant change in Ki67 + CMs in either sham or TAC hearts (Fig. 6c and Additional files 11: Fig. S7B and 12: S8) or gene expression between control and HP1γ KO (Fig. 6d, e).

### HP1γ loss does not alter gene expression profile after hemodynamic stress

**Fig. 7:**
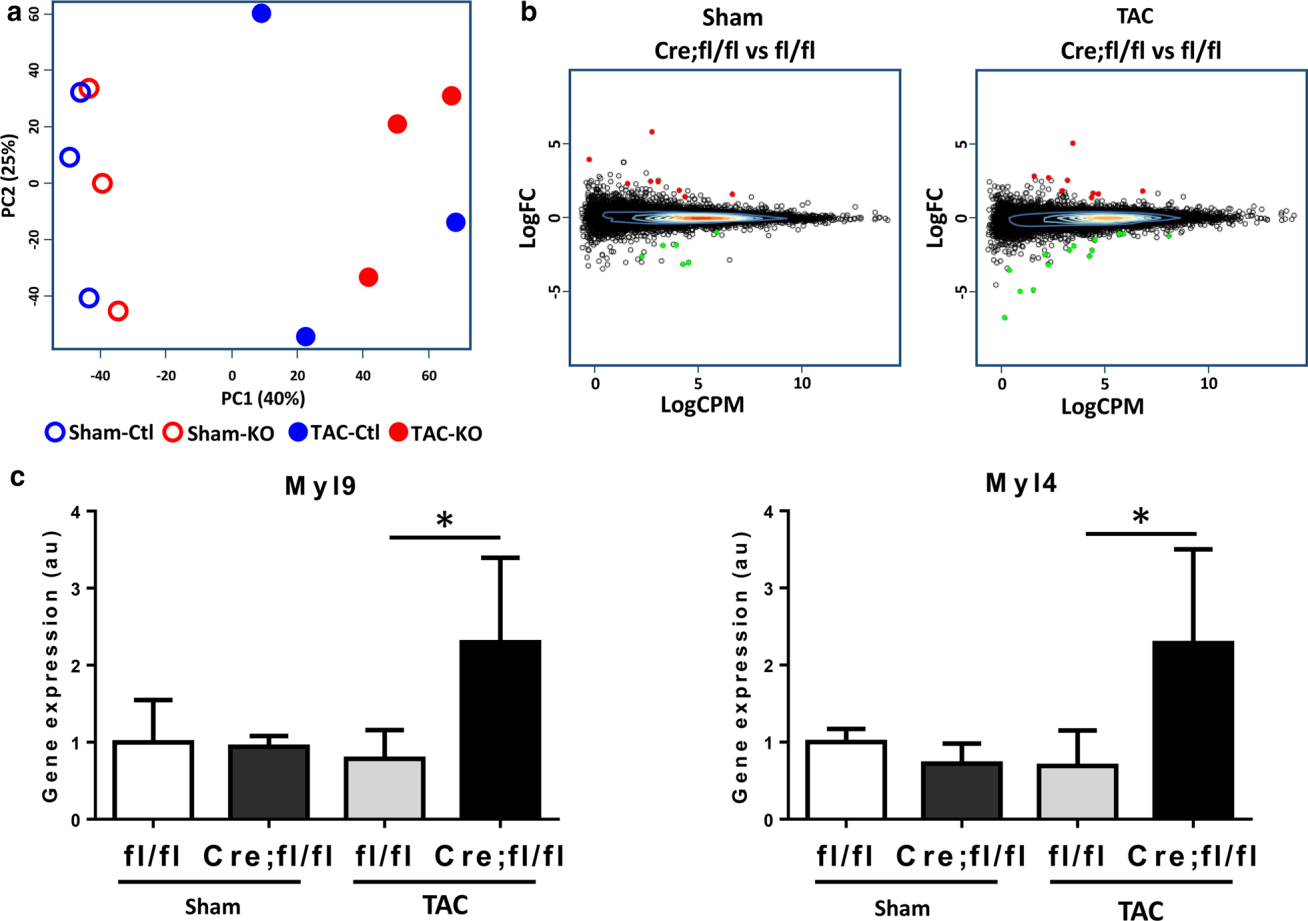
Effect of HP1γ KO on TAC-induced differential gene expression. **a** Principle component analysis. RNA-seq was performed purified CMs after TAC. Three independent biological samples were used for each group: Sham-control (fl/fl), Sham-HP1γ KO (Cre;fl/fl), TAC-control and TAC-HP1γ KO. **b** MA plot. *Y*-axis shows fold change of gene expression in HP1γ KO (Cre;fl/fl) vs control (fl/fl). *X*-axis shows average of count per million in all samples. Significantly, differentially expressed genes are shown in color (up regulation in red and down regulation in green). **c** RT-qPCR validation. Uniquely upregulated genes in HP1γ KO only after TAC were confirmed by RT-qPCR. *p < 0.05

Hypertrophic stress induces significant changes in the cardiac gene expression profile [42]. To test if HP1γ KO effected TAC-induced gene expression changes in CMs, we performed RNA-seq on HP1γ KO (Cre;fl/fl) and control (fl/fl) CMs 7 days after TAC (and Sham) operations. Principle component analysis showed a clear separation between sham group and TAC group on PC1 (Fig. 7a). However, there was no separation between genotypes in Sham or TAC operation, suggesting that there was a similar change in controls and HP1γ KO CM after TAC, and also that differences between control and HP1γ KO CMs were limited. Consistent with baseline RNA-seq results (Fig. 5) and PCA analysis (Fig. 7a), only 14 genes were detected as differentially expressed (FDR < 0.05) in Sham group, with 8 upregulation and 6 of down regulation in HP1γ KO compared to control (Fig. 7b; Additional file 2). 22 genes are detected as differentially expressed in HP1γ KO after TAC with 8 of upregulation and 14 of downregulation. We performed qPCR to validate these RNA-seq results. Consistent with baseline gene expression data (Fig. 5b), Cyp2b10 and Dlgap1 are significantly upregulated in HP1γ KO CMs in both sham and TAC operation (Additional file 13: figure S9). Two-way ANOVA analysis revealed a significant effect by HP1γ KO on these genes’ expression, but there was no effect by the operation nor the interaction between genotype and operation. Myl9 and Myl4 were detected by RNA-seq as significantly upregulated in HP1γ KO CMs only after TAC with FDR = 0.000061 and FDR = 0.0016, respectively. qPCR confirmed significant upregulation of Myl9 and Myl4 in HP1γ KO compared to control in TAC (Fig. 7c).

## Discussion

Our previous data suggested that H3K9me3 controlled cell cycle gene silencing in CMs. In this study, we tested whether H3K9me3 reader proteins, HP1s, could mediate this process. We focused on HP1γ for several reasons. Of the three HP1 isoforms HP1γ expression is the highest in CMs and HP1γ is the only isoform showing clear co-localization with H3K9me3 in ACMs (Fig. 1b, c and Additional file 1: Fig. S1). This finding suggested that HP1γ could be the critical isoform in CMs. We generated a cardiac-specific HP1γ deletion mouse model, which is the first report of a tissue-specific HP1γ KO. However, deleting HP1γ had minimal effect on global gene expression and no significant effect on cell cycle gene silencing, cardiac growth or function. This may be related to a compensatory effect of other HP1 family members, particularly HP1β, because HP1β expression was upregulated in HP1γ KO mice and its localization to the perinuclear region, which is normally occupied by HP1γ, was enhanced.

All HP1s contain two very well conserved domains, the CD and CSD, which allows different HP1 family proteins to form heterodimers with each other. Although slight binding preferences might exist between the HP1 family members, HP1s share many binding partner proteins [19, 21, 37, 38, 43]. These findings indicate that HP1s have overlapping roles and redundant functions, which is supported by this study where cardiac-specific HP1γ KO has a very mild phenotype. We also found that significant upregulation and altered localization of HP1β in HP1γ KO CMs (Fig. 3a, c and Additional file 6: Fig. S4B), suggesting compensatory upregulation. Upregulation of other HP1 isoforms was not reported in HP1γ hypomorphic or gene trapped HP1γ KO cells [23, 34, 35]. These findings suggest that compensatory upregulation could be cell-type specific. In contrast to our finding, indispensable roles for HP1s was reported in mice where there was constitutive disruption of HP1γ expression. Germline KO or a hypomorphic allele of HP1γ mouse showed defects in spermatogenesis and primordial germ cells [23, 34, 35]. Similarly, germ line deletion of HP1β showed aberrant cerebral cortex development and is perinatal lethal [44]. Thus, the necessity and role of each HP1 isoform appear to be cell type and context dependent. This study demonstrated that HP1γ is a dispensable, at least for normal cardiac growth and function.

HP1s have been shown to establish heterochromatin by mediating methylation of two of histone tails, H3K9me2/3 and H4K20me3. H3K9me2 and H3K9me3 are created by HMT G9a and Suv39h1, respectively [45, 46]. However, we did not detect any differences in global H3K9me2/3 level, indicating that HP1γ is dispensable to maintain H3K9me2/3 in CMs. In contrast, we found a significant reduction in H4K20me3 in HP1γ KO CM. Although both Suv420h1 and Suv420h2 can mediate H4K20me3 in vitro [38], Suv420h2 is the enzyme likely to establish H4K20me3 in vivo [47]. Suv420h2 has been shown to interact with all isoform of HP1s in both mouse and human [38, 48] and it is believed that targeting of Suv420h2 to chromatin is HP1 dependent, at least onto pericentromeric heterochromatin [49]. Recent work has shown that HP1β is likely to regulate targeting of Suv420h2 in mouse embryonic fibroblasts [50]. By contrast, we find that HP1γ is critical in regulating H4K20me3 levels and distribution in CMs. These data indicate that the regulation of H4K20me3 is likely to be cell-type specific and dependent upon HP1-isotype-specific interactions with Suv420h2.

Previous studies have reported a potential role of H3K9me3 and HP1s on cardiac hypertrophy including regulation of fetal cardiac gene expression [40, 51]. However, we found no effect of HP1γ deletion on cardiac gene expression (ANP, βMHC and αMHC) at baseline or after TAC (Figs. 4c and 6d). CM size and cell cycling was not changed in HP1γ KO mice either (Fig. 4b, c and Additional files 11: Fig. S7 and 12: S8). Although there was slight increase in heart mass in TAC-induced model, there was no difference of heart mass in isoproterenol-induced hypertrophy model in HP1γ KO mice (Additional file 14: Fig S10). These data suggest that HP1γ has a dispensable role in mediating cardiac hypertrophy.

Recent studies have shown that HP1s have a gene regulatory role, not only in gene silencing, but also activation [52, 53]. To our surprise, loss of HP1γ showed very moderate effect on gene expression in CMs (Fig. 5a, Additional file 9: Fig. S6 and Fig. 7a, b). Our RNA-seq and qPCR confirmed that Cyp2b10 and Dlgap1 were upregulated in HP1γ KO CMs (Fig. 5b and Additional file 13: Fig. S9). These genes are poorly expressed in CMs and consistent with this fact, ENCODE data show that promoters of these genes have limited accessibility, lack of active histone mark (H3K9ac) and no recruitment of Polymerase II in the heart [54], which suggest that these genes are heterochromatic. It is reasonable to hypothesize that HP1γ is suppressing these genes by HP1’s classic heterochromatin recruiting mechanism.

## Conclusions

We created a cardiac-specific HP1γ KO mouse model to test the role of HP1γ in cell cycle gene silencing and cardiac growth. To our knowledge, this is the first report examining the tissue- and isoform-specific role of HP1γ using a conditional KO system. We found that HP1γ has a significant role on H4K20me3 maintenance, although both HP1γ and H4K20me3 seem to be dispensable for cardiac cell cycle gene silencing and growth. Recent studies have reported the dispensability of classical heterochromatin regulators such as H3K9me2/3 [56] and DNA methylation [55] on gene silencing in vivo model, even though significant roles of these genes have been determined in vitro [16, 22, 57, 58]. This study highlights the complexity of epigenetic regulation and importance of examining the function of these regulators in vivo.

## Additional files

**Additional file 1: Fig. S1.** HP1 localization with H3K9me3. Cardiac myocytes were isolated from 10 week adult mice and HP1s (green) were co-immunostained with H3K9me3 (red) using specific antibodies. Heterochromatin was visualized by Hoechst staining (Blue). Scale bar indicates 5 μm

**Additional file 2.** DiffGene list after TAC.

**Additional file 3.** qPCR primer and antibody list.

**Additional file 4: Fig. S2.** **(A)** Primer design to detect deletion of critical exons on HP1γ gene using RT-PCR. HP1γ gene has 6 exons and start codon and inflame ATG are on exon 2 and exon 3, respectively. Forward primer and reverse primer are designed on exon 1 and 4, respectively, so that the primer pairs create long amplicon from normal HP1γ mRNA and 196 bp shorter amplicon from HP1γ KO mRNA by RT-PCR. **(B)** Organ specificity of Nkx2.5-Cre driven HP1γ KO. Total RNA was extracted from indicated organs from Cre;fl/fl animals as well as ACM from fl/fl and Cre;fl/fl. RT-PCR was performed using primer set described above. Representative pictures from repeated experiments is shown here.

**Additional file 5: Fig. S3.** Densitometry quantification of WB. Five biological replicates each genotype were used for densitometry quantification analysis. Protein expression level is show as relative expression value against WT. * *p* < 0.05.

**Additional file 6: Fig. S4.** Localization of heterochromatic histone marks **(A)** and HP1s **(B)**. 8-wk CMs from fl/fl control and KO (Cre;fl/fl) mice were isolated and stained with specific antibodies. Histone marks and HP1s are in red, cardiac troponin is in green and DNA is in blue. Scale bar indicates 10 μm.

**Additional file 7: Fig. S5.** **(A)** No difference in DNA damage marker in HP1γ KO CMs. Nuclear extracts were prepared from purified CM at 8wk and WB was performed using specific antibody against γH2AX. As a positive control of γH2AX induction, C2C12 cells were treated with doxorubicin (1 μM) for 6 h. Representative pictures of 5 biologically independent experiments are shown here. **(B and C)** No induction of cell cycle inhibitors was seen. Gene expression of p21 (marker for DNA damage and senescence) and p16 (marker for senescence) were measured by qPCR. p21 gene expression is normalized by S26 expression. RNA from irradiated mouse embryonic fibroblasts (iMEF) was used for p16 positive control. p16 gene expression is shown with Ct value of qPCR. p16 was undetectable in both control and HP1γ KO CMs (n = 4–5).

**Additional file 8: Table S1.** Comparison of cardiac function in HP1γ KO mouse. Echo cardiograms were taken at 8 weeks. All data are presented as mean ± SD. No significant difference was detected on parameters we measured by one-way ANOVA followed by multiple comparison.

**Additional file 9: Fig. S6.** Principle component analysis. RNA-seq was performed using purified CMs isolated from 8 week old mice. Three independent biological controls (WT, Cre, and fl/fl one of each) and HP1γ KO (Cre;fl/fl) samples were used.

**Additional file 10.** DiffGene list HP1γ KO 8wk baseline.

**Additional file 11: Fig. S7.** **(A)** Quantification of cardiac cross section area after TAC. More than 100 CM cross sections per animal were analyzed. Two-way ANOVA followed by multiple comparison was perform. TAC operation increased cross section area significantly; however, no interaction with genotype was detected. No significant difference was detected between genotype in Sham or TAC mice. **(B)** Quantification of cycling nuclear number in the heart. Heart sections were stained with Ki67 antibody and counted Ki67 positive nuclear number against total nuclear number. Since we could not find any cardiac nuclear positive for Ki67 in neither sham nor TAC condition, we estimated Ki67 positive nuclear number as cycling fibroblast number. Two-way ANOVA followed by multiple comparison was perform. TAC operation increased cycling fibroblast number significantly; however, no interaction with genotype was detected.

**Additional file 12: Fig. S8.** No difference in cycling CM in HP1γ KO heart. TAC or Sham surgeries were performed at 10–12 weeks and hearts harvested 1 week after operations. Heart tissues were stained for Ki67 (white), Hoechst (red) and WGA (green). Scale bar indicates 100 μm.

**Additional file 13: Fig. S9.** Upregulated genes in HP1γ KO in both sham and TAC. RT-qPCR was performed to confirm differential gene expressed detected by RNA-seq. Two-way ANOVA followed by multiple comparison demonstrated that genotype has significant effect on Cyp2b10 and Dlgap1 expression, but not TAC operation or interaction between genotype and TAC operation.

**Additional file 14: Fig. S10.** Effect of HP1γ KO on ISO-induced heart growth. ISO (5 μg/g) was injected subcutaneously once a day for 6 days and hearts were harvested. Same volume of saline was injected as a vehicle control. Since there was no difference of HW normalized by BW at base line, all genotype of animals with vehicle injection are grouped as a vehicle control group. Upon ISO treatment, all genotype showed a significant increase in HW/BW compared to vehicle control; however, there was no difference between genotype, indicating that HP1γ KO doesn’t have a significant effect on ISO-induced heart growth. * *p* < 0.05 vs vehicle control.

## Abbreviations

CM: cardiac myocyte
ACM: adult cardiac myocyte
SAHF: senescence-associated heterochromatin foci
H3K9me3: tri-methyl lysine 9 of histone H3
H4K20me3: tri-methyl lysine 20 of histone H4
HP1: heterochromatin protein 1
HMT: histone methyl transferase
RB: retinoblastoma protein
CD: chromo domain
CSD: chromo shadow domain
ECM: embryonic cardiac myocyte
HW: heart weight
BW: body weight
TAC: transverse aortic constriction

## References

[1] Ahuja P, Sdek P, MacLellan WR (2007). Cardiac myocyte cell cycle control in development, disease, and regeneration. Physiol Rev.

[2] Leone M, Magadum A, Engel FB (2015). Cardiomyocyte proliferation in cardiac development and regeneration: a guide to methodologies and interpretations. Am J Physiol Heart Circ Physiol.

[3] Oyama K, El-Nachef D, Zhang Y, Sdek P, MacLellan WR (2014). Epigenetic regulation of cardiac myocyte differentiation. Front Genet.

[4] Sdek P, Zhao P, Wang Y, Huang CJ, Ko CY, Butler PC, Weiss JN, MacLellan WR (2011). Rb and p130 control cell cycle gene silencing to maintain the postmitotic phenotype in cardiac myocytes. J Cell Biol.

[5] Narita M, Nunez S, Heard E, Narita M, Lin AW, Hearn SA, Spector DL, Hannon GJ, Lowe SW (2003). Rb-mediated heterochromatin formation and silencing of E2F target genes during cellular senescence. Cell.

[6] Zhang R, Poustovoitov MV, Ye X, Santos HA, Chen W, Daganzo SM, Erzberger JP, Serebriiskii IG, Canutescu AA, Dunbrack RL, Pehrson JR, Berger JM, Kaufman PD, Adams PD (2005). Formation of MacroH2A-containing senescence-associated heterochromatin foci and senescence driven by ASF1a and HIRA. Dev Cell.

[7] Zhang R, Chen W, Adams PD (2007). Molecular dissection of formation of senescence-associated heterochromatin foci. Mol Cell Biol.

[8] Henley SA, Dick FA (2012). The retinoblastoma family of proteins and their regulatory functions in the mammalian cell division cycle. Cell Div.

[9] Dick FA, Rubin SM (2013). Molecular mechanisms underlying RB protein function. Nat Rev Mol Cell Biol.

[10] Nielsen SJ, Schneider R, Bauer UM, Bannister AJ, Morrison A, O’Carroll D, Firestein R, Cleary M, Jenuwein T, Herrera RE, Kouzarides T (2001). Rb targets histone H3 methylation and HP1 to promoters. Nature.

[11] Magnaghi-Jaulin L, Groisman R, Naguibneva I, Robin P, Lorain S, Le Villain JP, Troalen F, Trouche D, Harel-Bellan A (1998). Retinoblastoma protein represses transcription by recruiting a histone deacetylase. Nature.

[12] Vandel L, Nicolas E, Vaute O, Ferreira R, Ait-Si-Ali S, Trouche D (2001). Transcriptional repression by the retinoblastoma protein through the recruitment of a histone methyltransferase. Mol Cell Biol.

[13] El-Nachef D, Oyama K, Wu Y-Y, Liu Yonggang, Zhang Y, MacLellan WR (2016). Epigenetic control of adult cardiac myocyte proliferation [abstract]. Circulation.

[14] Eissenberg JC, James TC, Foster-Hartnett DM, Hartnett T, Ngan V, Elgin SC (1990). Mutation in a heterochromatin-specific chromosomal protein is associated with suppression of position-effect variegation in *Drosophila melanogaster*. Proc Natl Acad Sci USA.

[15] James TC, Elgin SC (1986). Identification of a nonhistone chromosomal protein associated with heterochromatin in *Drosophila melanogaster* and its gene. Mol Cell Biol.

[16] Bannister AJ, Zegerman P, Partridge JF, Miska EA, Thomas JO, Allshire RC, Kouzarides T (2001). Selective recognition of methylated lysine 9 on histone H3 by the HP1 chromo domain. Nature.

[17] Cowieson NP, Partridge JF, Allshire RC, McLaughlin PJ (2000). Dimerisation of a chromo shadow domain and distinctions from the chromodomain as revealed by structural analysis. Curr Biol.

[18] Lachner M, O’Carroll D, Rea S, Mechtler K, Jenuwein T (2001). Methylation of histone H3 lysine 9 creates a binding site for HP1 proteins. Nature.

[19] Raurell-Vila H, Bosch-Presegue L, Gonzalez J, Kane-Goldsmith N, Casal C, Brown JP, Marazuela-Duque A, Singh PB, Serrano L, Vaquero A (2017). An HP1 isoform-specific feedback mechanism regulates Suv39h1 activity under stress conditions. Epigenetics.

[20] Smothers JF, Henikoff S (2000). The HP1 chromo shadow domain binds a consensus peptide pentamer. Curr Biol.

[21] Yamamoto K, Sonoda M (2003). Self-interaction of heterochromatin protein 1 is required for direct binding to histone methyltransferase, SUV39H1. Biochem Biophys Res Commun.

[22] Hathaway NA, Bell O, Hodges C, Miller EL, Neel DS, Crabtree GR (2012). Dynamics and memory of heterochromatin in living cells. Cell.

[23] Brown JP, Bullwinkel J, Baron-Luhr B, Billur M, Schneider P, Winking H, Singh PB (2010). HP1gamma function is required for male germ cell survival and spermatogenesis. Epigenetics Chromatin.

[24] Farley FW, Soriano P, Steffen LS, Dymecki SM (2000). Widespread recombinase expression using FLPeR (flipper) mice. Genesis.

[25] Moses KA, DeMayo F, Braun RM, Reecy JL, Schwartz RJ (2001). Embryonic expression of an Nkx2-5/Cre gene using ROSA26 reporter mice. Genesis.

[26] Tarnavski O, McMullen JR, Schinke M, Nie Q, Kong S, Izumo S (2004). Mouse cardiac surgery: comprehensive techniques for the generation of mouse models of human diseases and their application for genomic studies. Physiol Genomics.

[27] Weldy CS, Liu Y, Liggitt HD, Chin MT (2014). In utero exposure to diesel exhaust air pollution promotes adverse intrauterine conditions, resulting in weight gain, altered blood pressure, and increased susceptibility to heart failure in adult mice. PLoS ONE.

[28] Kim D, Pertea G, Trapnell C, Pimentel H, Kelley R, Salzberg SL (2013). TopHat2: accurate alignment of transcriptomes in the presence of insertions, deletions and gene fusions. Genome Biol.

[29] Trapnell C, Williams BA, Pertea G, Mortazavi A, Kwan G, van Baren MJ, Salzberg SL, Wold BJ, Pachter L (2010). Transcript assembly and quantification by RNA-Seq reveals unannotated transcripts and isoform switching during cell differentiation. Nat Biotechnol.

[30] Anders S, Pyl PT, Huber W (2015). HTSeq–a Python framework to work with high-throughput sequencing data. Bioinformatics.

[31] Robinson MD, McCarthy DJ, Smyth GK (2010). edgeR: a Bioconductor package for differential expression analysis of digital gene expression data. Bioinformatics.

[32] McCarthy DJ, Chen Y, Smyth GK (2012). Differential expression analysis of multifactor RNA-Seq experiments with respect to biological variation. Nucleic Acids Res.

[33] Hilwig I, Gropp A (1973). Decondensation of constitutive heterochromatin in L cell chromosomes by a benzimidazole compound (“33258 Hoechst”). Exp Cell Res.

[34] Takada Y, Naruse C, Costa Y, Shirakawa T, Tachibana M, Sharif J, Kezuka-Shiotani F, Kakiuchi D, Masumoto H, Shinkai Y, Ohbo K, Peters AH, Turner JM, Asano M, Koseki H (2011). HP1gamma links histone methylation marks to meiotic synapsis in mice. Development.

[35] Abe K, Naruse C, Kato T, Nishiuchi T, Saitou M, Asano M (2011). Loss of heterochromatin protein 1 gamma reduces the number of primordial germ cells via impaired cell cycle progression in mice. Biol Reprod.

[36] Maison C, Almouzni G (2004). HP1 and the dynamics of heterochromatin maintenance. Nat Rev Mol Cell Biol.

[37] Chin HG, Esteve PO, Pradhan M, Benner J, Patnaik D, Carey MF, Pradhan S (2007). Automethylation of G9a and its implication in wider substrate specificity and HP1 binding. Nucleic Acids Res.

[38] Schotta G, Lachner M, Sarma K, Ebert A, Sengupta R, Reuter G, Reinberg D, Jenuwein T (2004). A silencing pathway to induce H3-K9 and H4-K20 trimethylation at constitutive heterochromatin. Genes Dev.

[39] Dambacher S, Hahn M, Schotta G (2013). The compact view on heterochromatin. Cell Cycle.

[40] Hohl M, Wagner M, Reil JC, Muller SA, Tauchnitz M, Zimmer AM, Lehmann LH, Thiel G, Bohm M, Backs J, Maack C (2013). HDAC4 controls histone methylation in response to elevated cardiac load. J Clin Investig.

[41] Metsalu T, Vilo J (2015). ClustVis: a web tool for visualizing clustering of multivariate data using Principal Component Analysis and heatmap. Nucleic Acids Res.

[42] Greco CM, Kunderfranco P, Rubino M, Larcher V, Carullo P, Anselmo A, Kurz K, Carell T, Angius A, Latronico MV, Papait R, Condorelli G (2016). DNA hydroxymethylation controls cardiomyocyte gene expression in development and hypertrophy. Nat Commun.

[43] Rosnoblet C, Vandamme J, Volkel P, Angrand PO (2011). Analysis of the human HP1 interactome reveals novel binding partners. Biochem Biophys Res Commun.

[44] Aucott R, Bullwinkel J, Yu Y, Shi W, Billur M, Brown JP, Menzel U, Kioussis D, Wang G, Reisert I, Weimer J, Pandita RK, Sharma GG, Pandita TK, Fundele R, Singh PB (2008). HP1-beta is required for development of the cerebral neocortex and neuromuscular junctions. J Cell Biol.

[45] Peters AH, Kubicek S, Mechtler K, O’Sullivan RJ, Derijck AA, Perez-Burgos L, Kohlmaier A, Opravil S, Tachibana M, Shinkai Y, Martens JH, Jenuwein T (2003). Partitioning and plasticity of repressive histone methylation states in mammalian chromatin. Mol Cell.

[46] Tachibana M, Sugimoto K, Nozaki M, Ueda J, Ohta T, Ohki M, Fukuda M, Takeda N, Niida H, Kato H, Shinkai Y (2002). G9a histone methyltransferase plays a dominant role in euchromatic histone H3 lysine 9 methylation and is essential for early embryogenesis. Genes Dev.

[47] Schotta G, Sengupta R, Kubicek S, Malin S, Kauer M, Callen E, Celeste A, Pagani M, Opravil S, De La Rosa-Velazquez IA, Espejo A, Bedford MT, Nussenzweig A, Busslinger M, Jenuwein T (2008). A chromatin-wide transition to H4K20 monomethylation impairs genome integrity and programmed DNA rearrangements in the mouse. Genes Dev.

[48] Souza PP, Volkel P, Trinel D, Vandamme J, Rosnoblet C, Heliot L, Angrand PO (2009). The histone methyltransferase SUV420H2 and Heterochromatin Proteins HP1 interact but show different dynamic behaviours. BMC Cell Biol.

[49] Tsang LW, Hu N, Underhill DA (2010). Comparative analyses of SUV420H1 isoforms and SUV420H2 reveal differences in their cellular localization and effects on myogenic differentiation. PLoS ONE.

[50] Bosch-Presegue L, Raurell-Vila H, Thackray JK, Gonzalez J, Casal C, Kane-Goldsmith N, Vizoso M, Brown JP, Gomez A, Ausio J, Zimmermann T, Esteller M, Schotta G, Singh PB, Serrano L, Vaquero A (2017). Mammalian HP1 isoforms have specific roles in heterochromatin structure and organization. Cell Rep.

[51] Zhang QJ, Chen HZ, Wang L, Liu DP, Hill JA, Liu ZP (2011). The histone trimethyllysine demethylase JMJD2A promotes cardiac hypertrophy in response to hypertrophic stimuli in mice. J Clin Investig.

[52] Vakoc CR, Mandat SA, Olenchock BA, Blobel GA (2005). Histone H3 lysine 9 methylation and HP1gamma are associated with transcription elongation through mammalian chromatin. Mol Cell.

[53] Mateescu B, Bourachot B, Rachez C, Ogryzko V, Muchardt C (2008). Regulation of an inducible promoter by an HP1beta-HP1gamma switch. EMBO Rep.

[54] ENCODE Project Consortium (2012). An integrated encyclopedia of DNA elements in the human genome. Nature.

[55] Nuhrenberg TG, Hammann N, Schnick T, Preissl S, Witten A, Stoll M, Gilsbach R, Neumann FJ, Hein L (2015). Cardiac Myocyte De Novo DNA Methyltransferases 3a/3b Are Dispensable for Cardiac Function and Remodeling after Chronic Pressure Overload in Mice. PLoS ONE.

[56] Zeller P, Padeken J (2016). van SR, Kalck V, Tijsterman M, Gasser SM: Histone H3K9 methylation is dispensable for Caenorhabditis elegans development but suppresses RNA:DNA hybrid-associated repeat instability. Nat Genet.

[57] Vojta A, Dobrinic P, Tadic V, Bockor L, Korac P, Julg B, Klasic M, Zoldos V (2016). Repurposing the CRISPR-Cas9 system for targeted DNA methylation. Nucleic Acids Res.

[58] Canzio D, Chang EY, Shankar S, Kuchenbecker KM, Simon MD, Madhani HD, Narlikar GJ, Al-Sady B (2011). Chromodomain-mediated oligomerization of HP1 suggests a nucleosome-bridging mechanism for heterochromatin assembly. Mol Cell.

