# Supplementary figures and images for: Deletion of HP1γ in cardiac myocytes affects H4K20me3 levels but does not impact cardiac growth
Source: Epigenetics Chromatin. 2018 Apr 17;11:18. doi: 10.1186/s13072-018-0187-z (PMC5905015; doi:10.1186/s13072-018-0187-z)

Additional file 1: Fig. S1

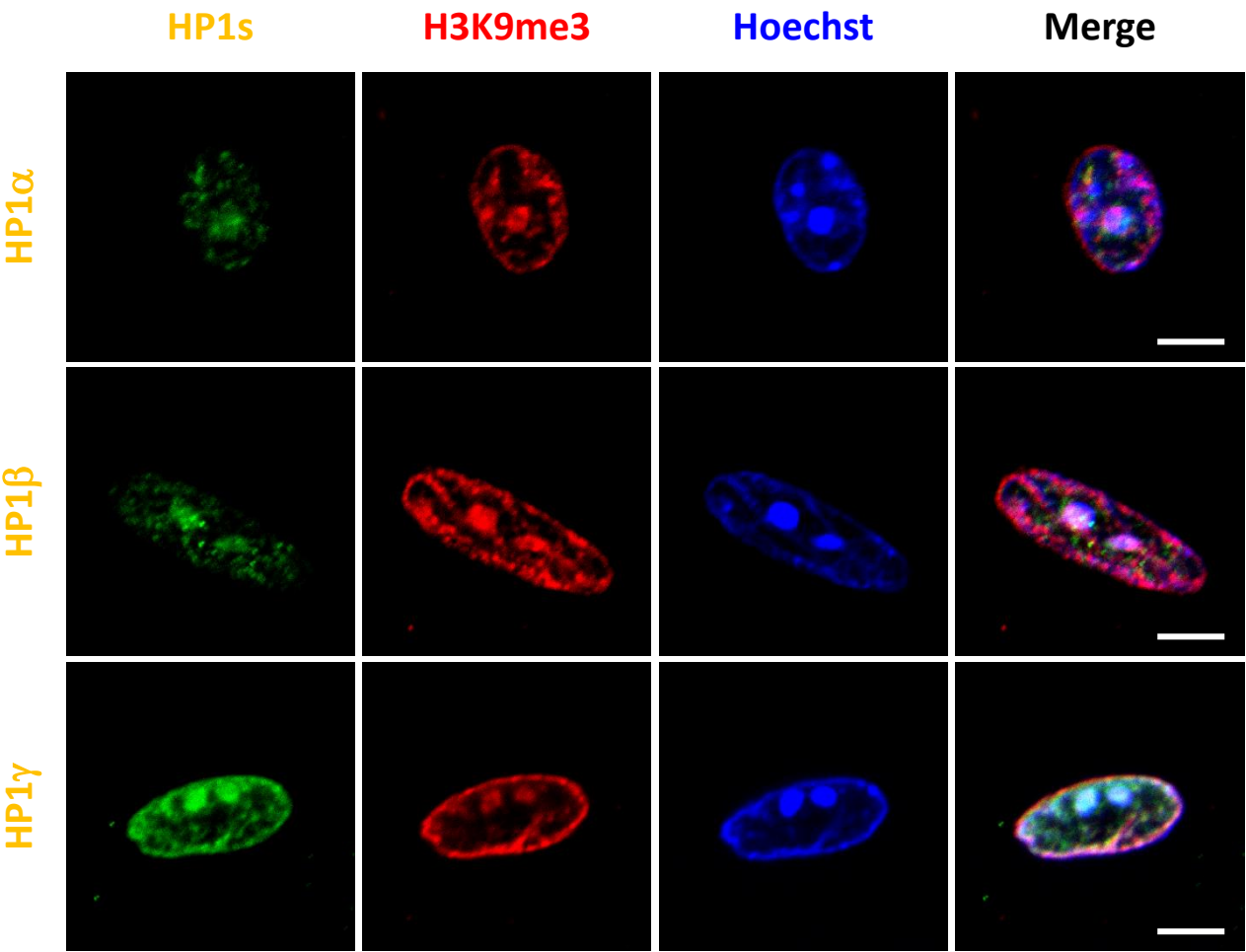

Supplement: Supplementary file 1 — Additional file 1: Fig. S1. HP1 localization with H3K9me3. Cardiac myocytes were isolated from 10 week adult mice and HP1s (green) were co-immunostained with H3K9me3 (red) using specific antibodies. Heterochromatin was visualized by Hoechst staining (Blue). Scale bar indicates 5 μm [file 13072_2018_187_MOESM1_ESM.pdf]

Additional file 3: Fig. S2

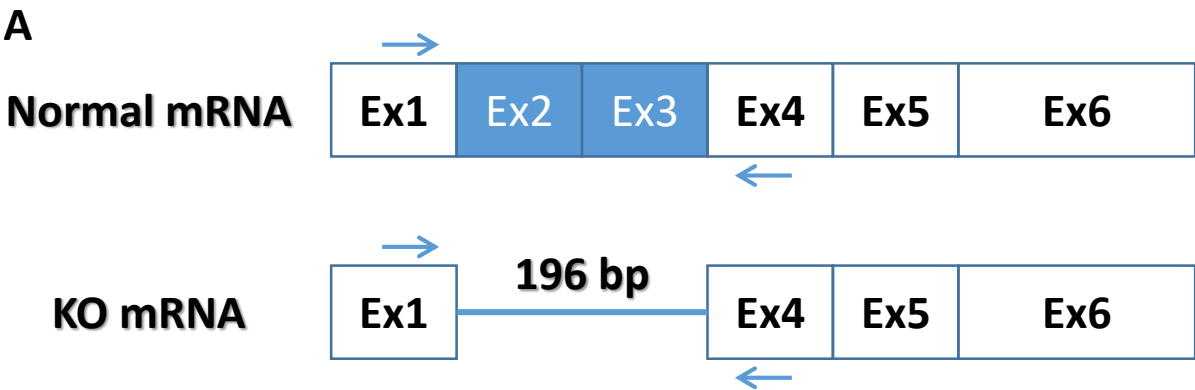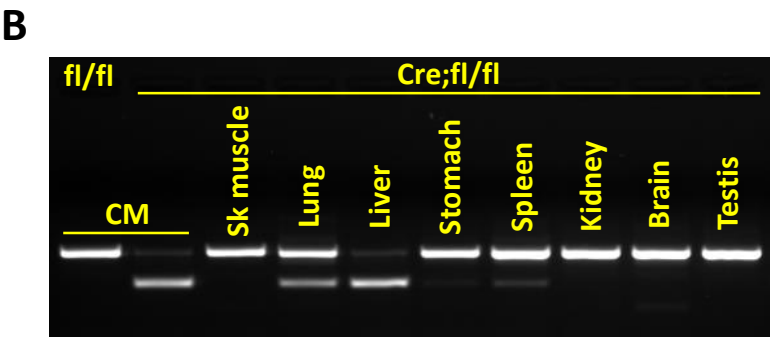

Supplement: Supplementary file 4 — Additional file 4: Fig. S2. (A) Primer design to detect deletion of critical exons on HP1γ gene using RT-PCR. HP1γ gene has 6 exons and start codon and inflame ATG are on exon 2 and exon 3, respectively. Forward primer and reverse primer are designed on exon 1 and 4, respectively, so that the primer pairs create long amplicon from normal HP1γ mRNA and 196 bp shorter amplicon from HP1γ KO mRNA by RT-PCR. (B) Organ specificity of Nkx2.5-Cre driven HP1γ KO. Total RNA was extracted from indicated organs from Cre;fl/fl animals as well as ACM from fl/fl and Cre;fl/fl. RT-PCR was performed using primer set described above. Representative pictures from repeated experiments is shown here. [file 13072_2018_187_MOESM4_ESM.pdf]

Additional file 4: Fig. S3

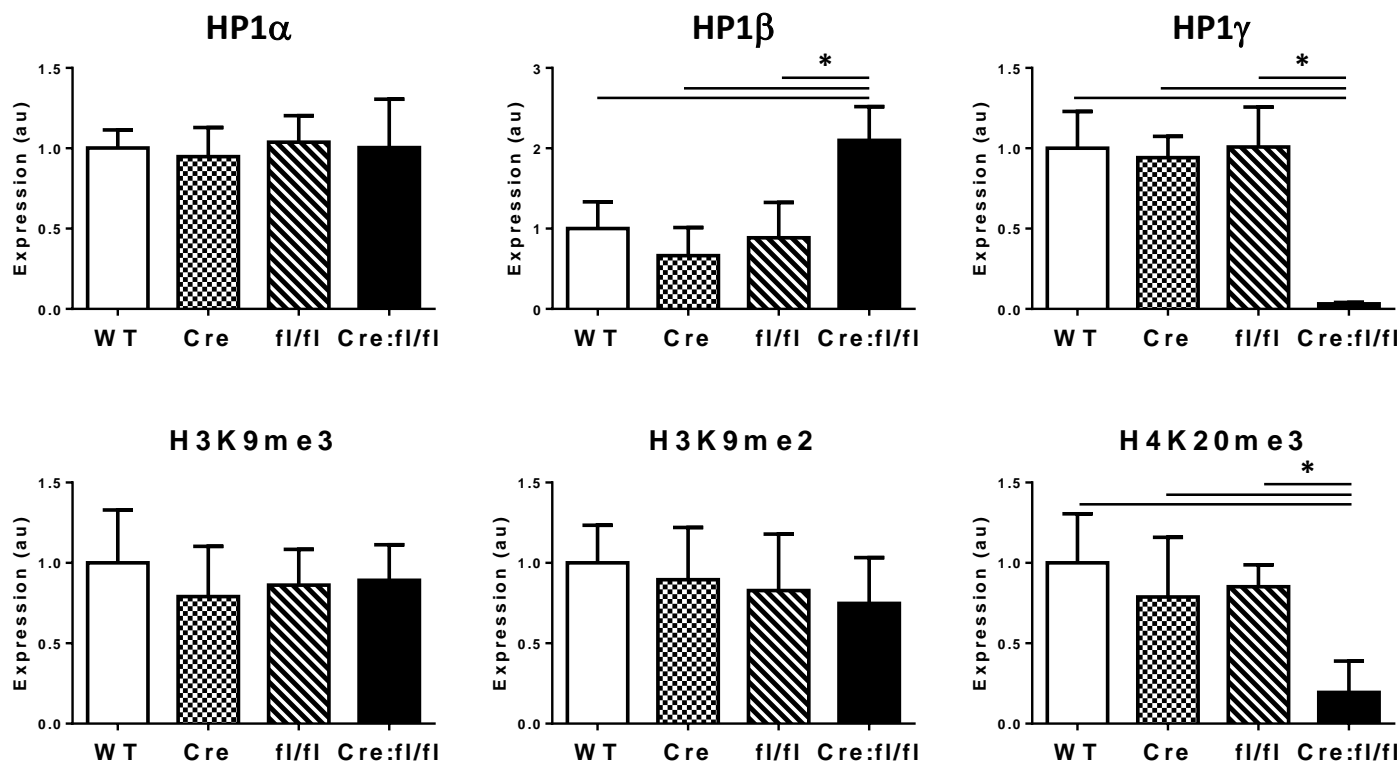

Supplement: Supplementary file 5 — Additional file 5: Fig. S3. Densitometry quantification of WB. Five biological replicates each genotype were used for densitometry quantification analysis. Protein expression level is show as relative expression value against WT. * p < 0.05. [file 13072_2018_187_MOESM5_ESM.pdf]

Additional file 5: Fig. S4A

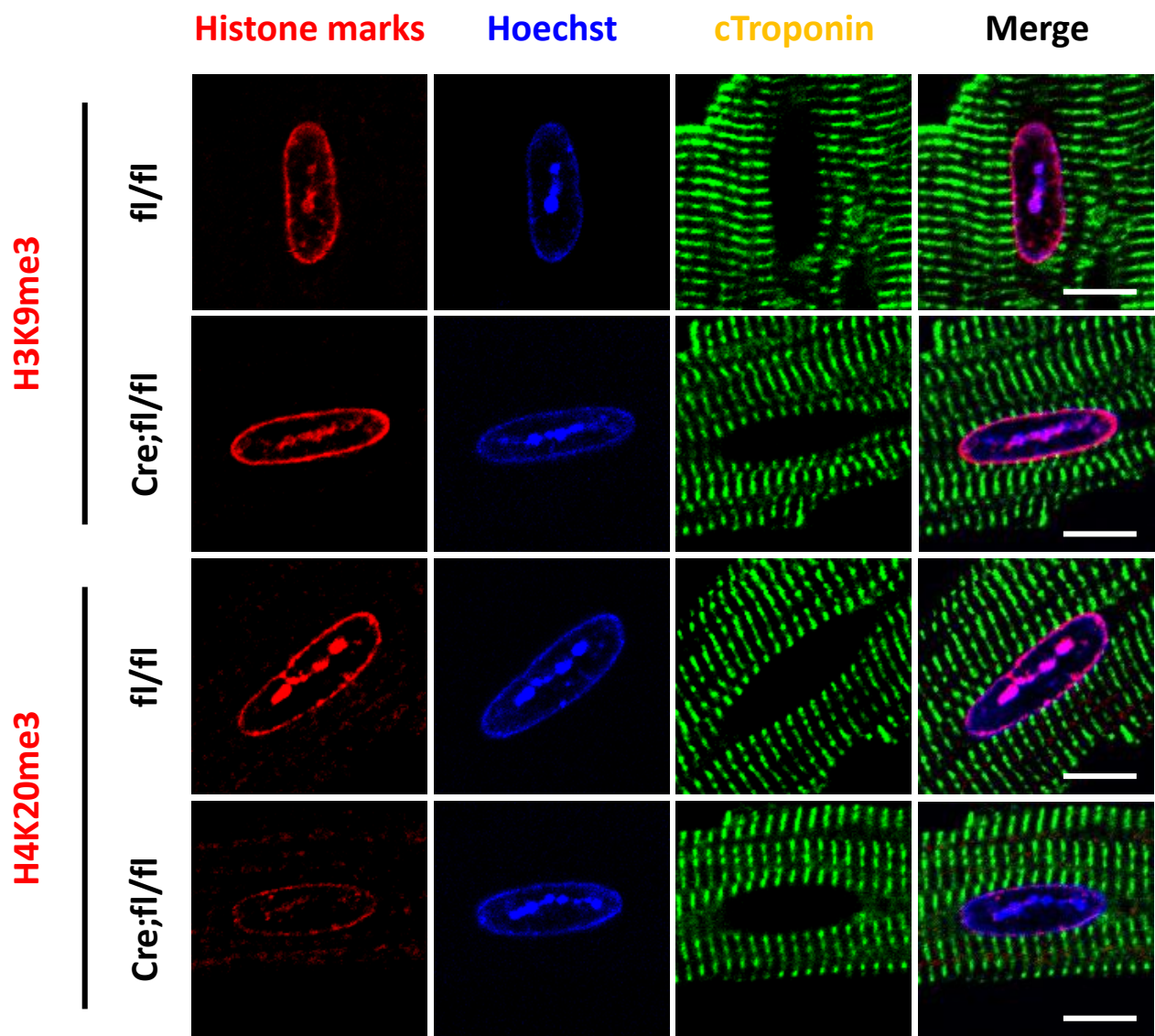

Additional file 5: Fig. S4B

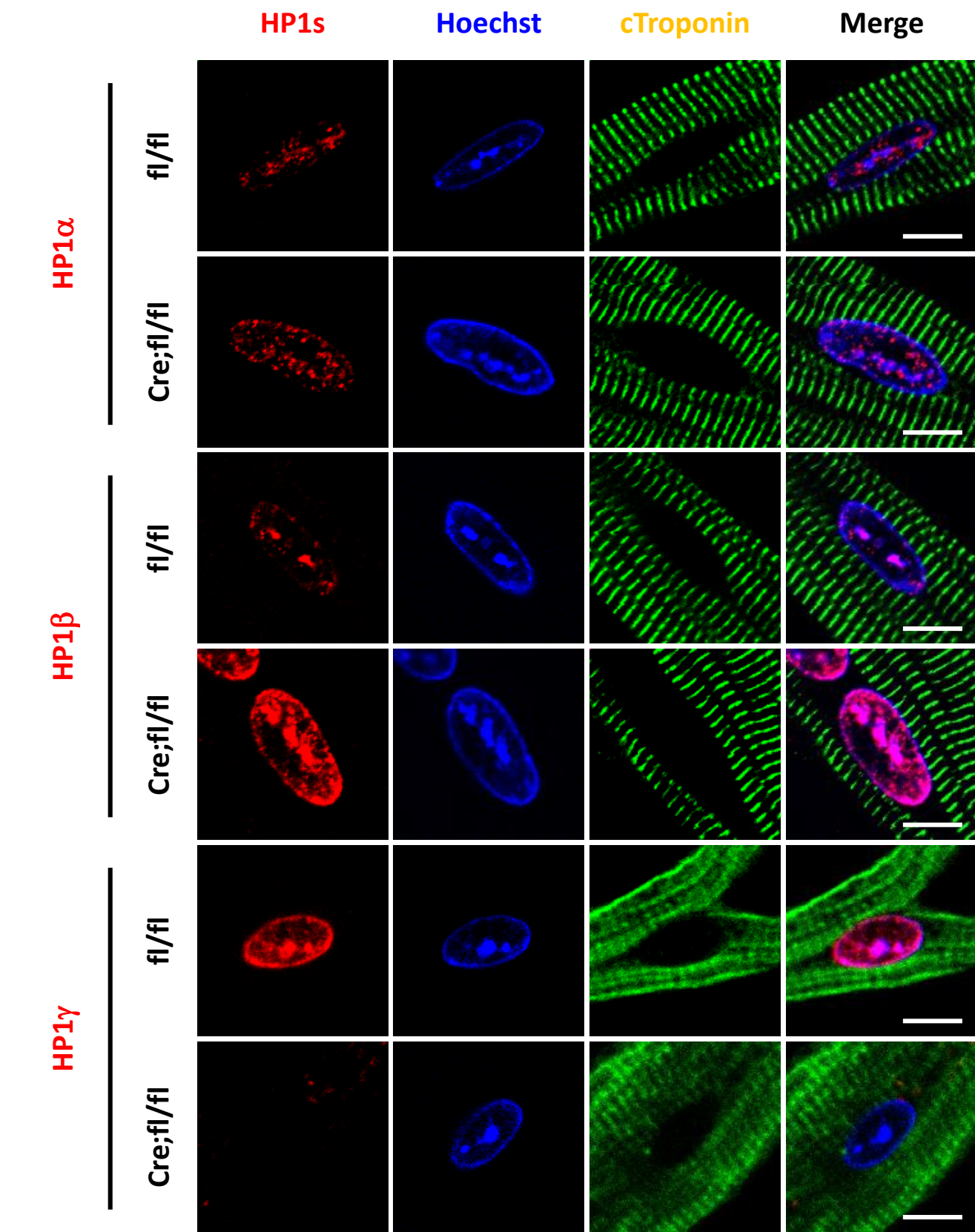

Supplement: Supplementary file 6 — Additional file 6: Fig. S4. Localization of heterochromatic histone marks (A) and HP1s (B). 8-wk CMs from fl/fl control and KO (Cre;fl/fl) mice were isolated and stained with specific antibodies. Histone marks and HP1s are in red, cardiac troponin is in green and DNA is in blue. Scale bar indicates 10 μm. [file 13072_2018_187_MOESM6_ESM.pdf]

Additional file 6: Fig. S5

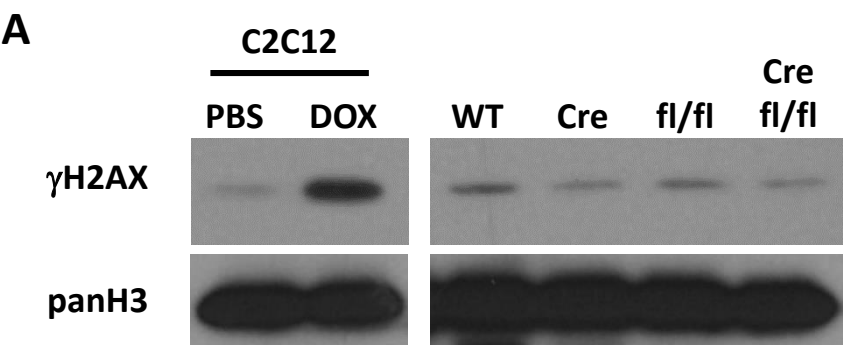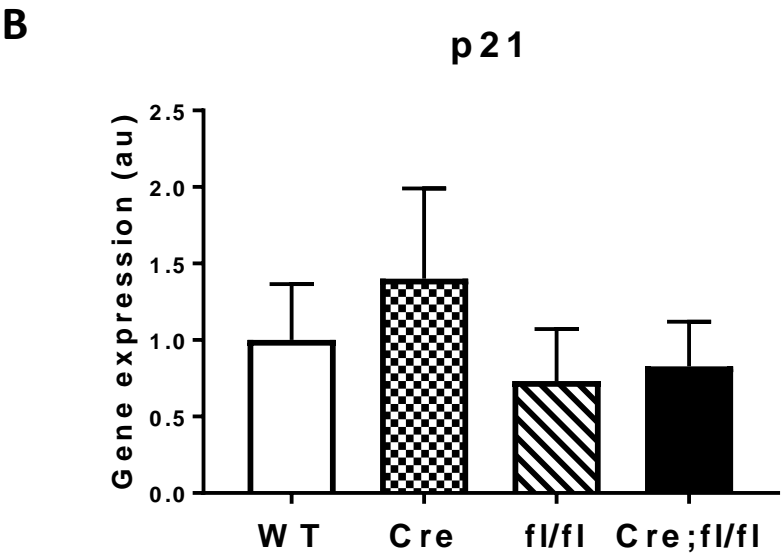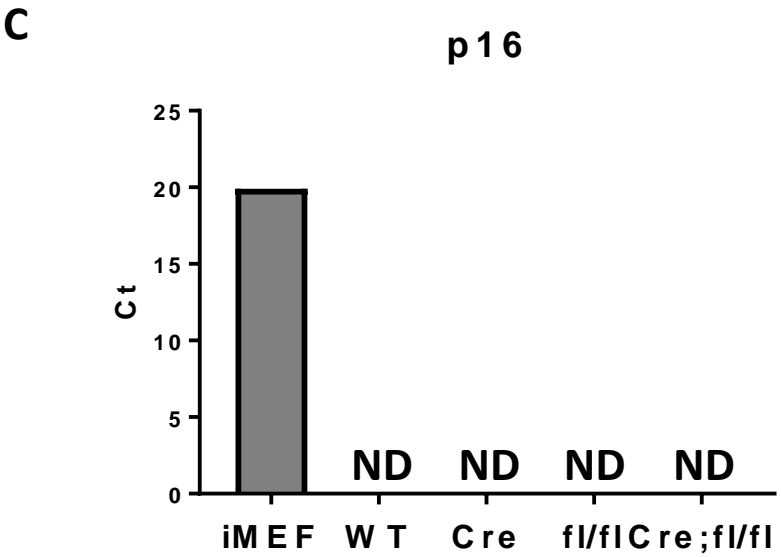

Supplement: Supplementary file 7 — Additional file 7: Fig. S5. (A) No difference in DNA damage marker in HP1γ KO CMs. Nuclear extracts were prepared from purified CM at 8wk and WB was performed using specific antibody against γH2AX. As a positive control of γH2AX induction, C2C12 cells were treated with doxorubicin (1 μM) for 6 h. Representative pictures of 5 biologically independent experiments are shown here. (B and C) No induction of cell cycle inhibitors was seen. Gene expression of p21 (marker for DNA damage and senescence) and p16 (marker for senescence) were measured by qPCR. p21 gene expression is normalized by S26 expression. RNA from irradiated mouse embryonic fibroblasts (iMEF) was used for p16 positive control. p16 gene expression is shown with Ct value of qPCR. p16 was undetectable in both control and HP1γ KO CMs (n = 4–5). [file 13072_2018_187_MOESM7_ESM.pdf]

Additional file8: Fig. S6

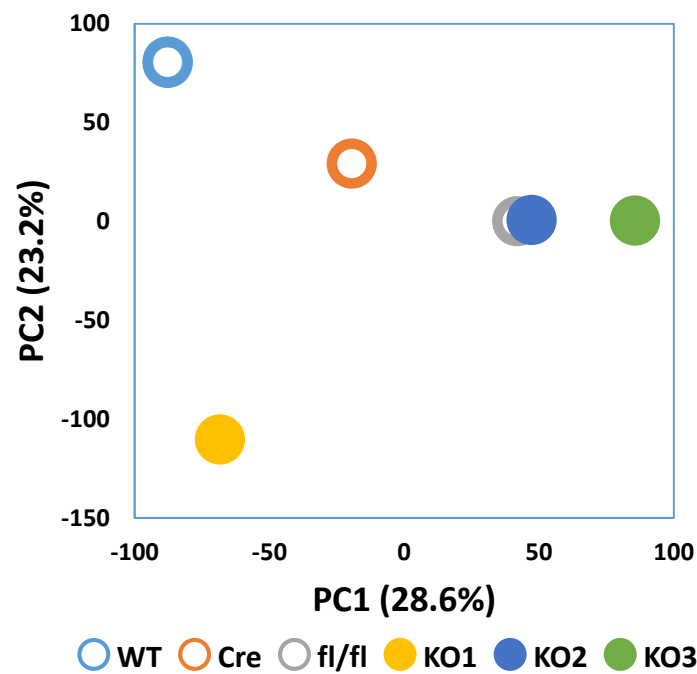

Supplement: Supplementary file 9 — Additional file 9: Fig. S6. Principle component analysis. RNA-seq was performed using purified CMs isolated from 8 week old mice. Three independent biological controls (WT, Cre, and fl/fl one of each) and HP1γ KO (Cre;fl/fl) samples were used. [file 13072_2018_187_MOESM9_ESM.pdf]

Additional file 10, Fig. S7

A

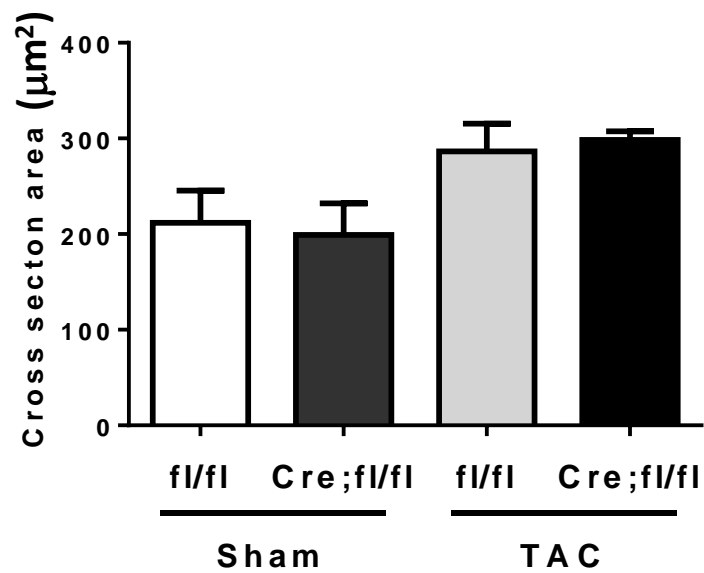

B

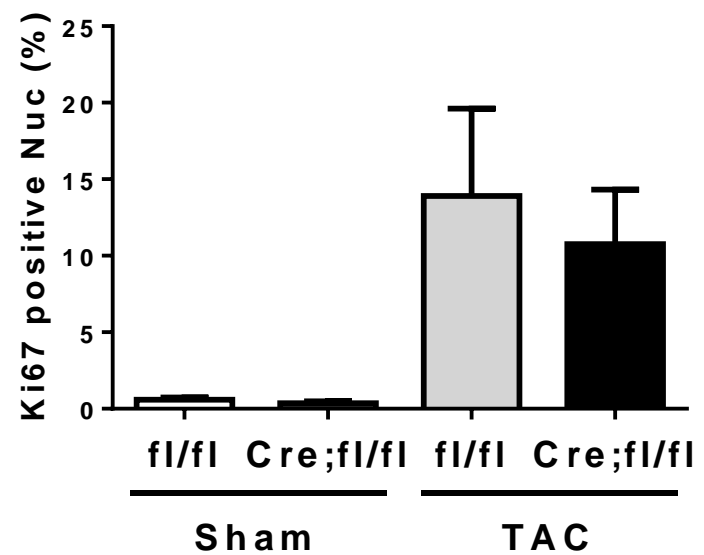

Supplement: Supplementary file 11 — Additional file 11: Fig. S7. (A) Quantification of cardiac cross section area after TAC. More than 100 CM cross sections per animal were analyzed. Two-way ANOVA followed by multiple comparison was perform. TAC operation increased cross section area significantly; however, no interaction with genotype was detected. No significant difference was detected between genotype in Sham or TAC mice. (B) Quantification of cycling nuclear number in the heart. Heart sections were stained with Ki67 antibody and counted Ki67 positive nuclear number against total nuclear number. Since we could not find any cardiac nuclear positive for Ki67 in neither sham nor TAC condition, we estimated Ki67 positive nuclear number as cycling fibroblast number. Two-way ANOVA followed by multiple comparison was perform. TAC operation increased cycling fibroblast number significantly; however, no interaction with genotype was detected. [file 13072_2018_187_MOESM11_ESM.pdf]

Additional file 11: Fig. S8

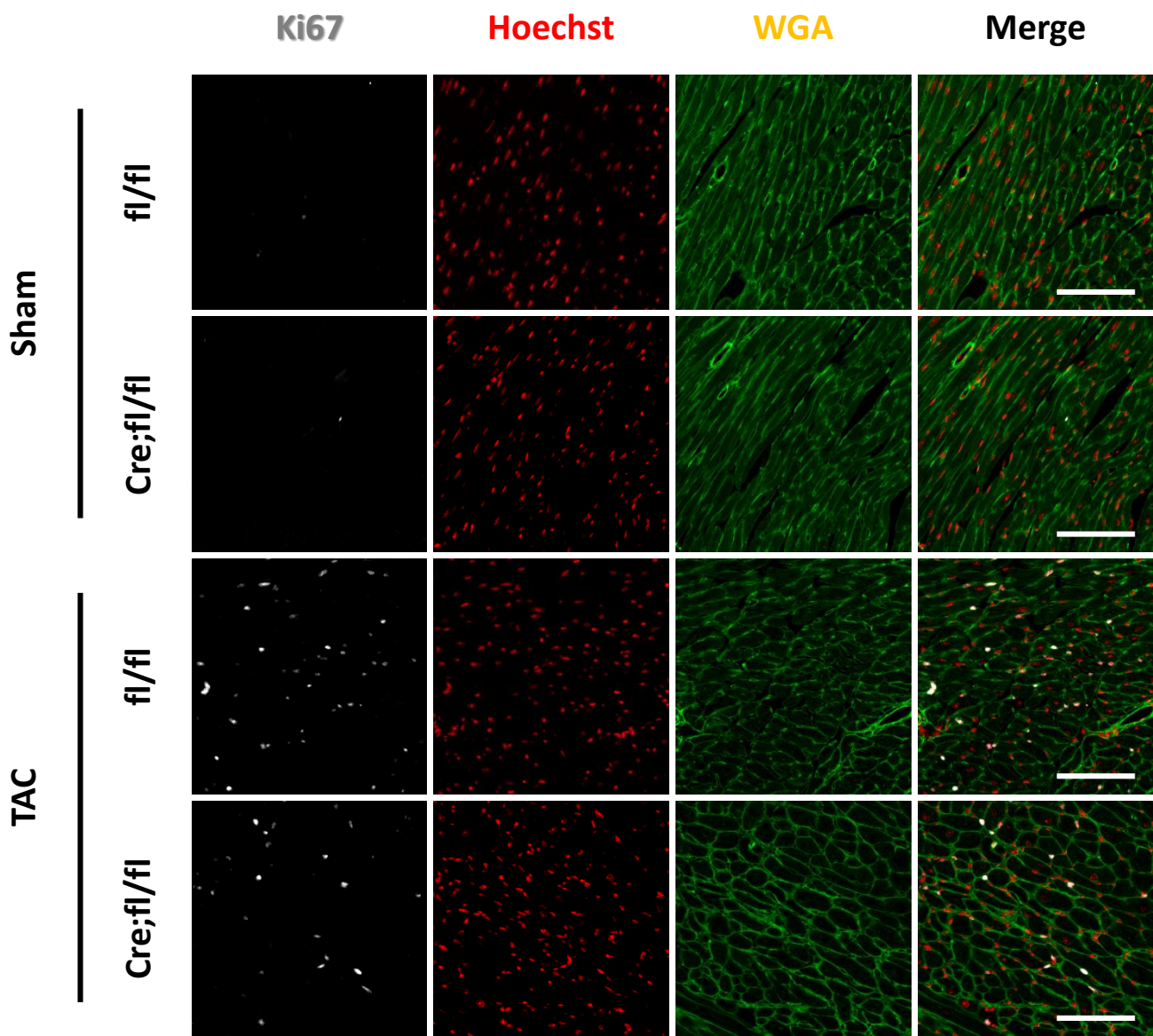

Supplement: Supplementary file 12 — Additional file 12: Fig. S8. No difference in cycling CM in HP1γ KO heart. TAC or Sham surgeries were performed at 10–12 weeks and hearts harvested 1 week after operations. Heart tissues were stained for Ki67 (white), Hoechst (red) and WGA (green). Scale bar indicates 100 μm. [file 13072_2018_187_MOESM12_ESM.pdf]

Additional file 13: Fig. S9

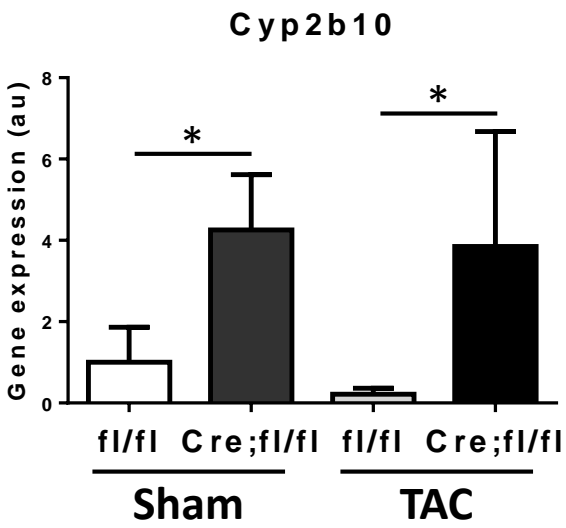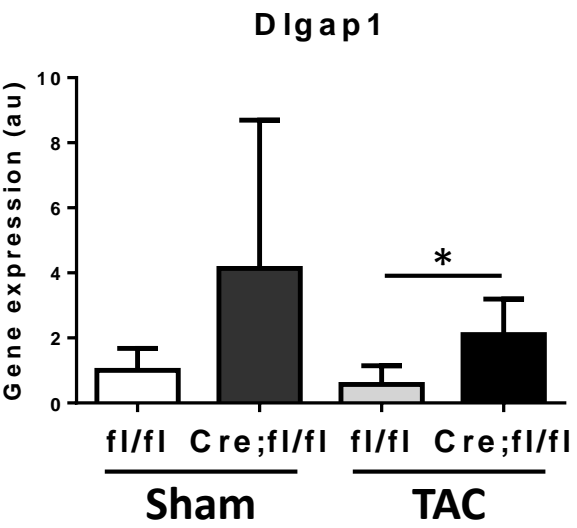

Supplement: Supplementary file 13 — Additional file 13: Fig. S9. Upregulated genes in HP1γ KO in both sham and TAC. RT-qPCR was performed to confirm differential gene expressed detected by RNA-seq. Two-way ANOVA followed by multiple comparison demonstrated that genotype has significant effect on Cyp2b10 and Dlgap1 expression, but not TAC operation or interaction between genotype and TAC operation. [file 13072_2018_187_MOESM13_ESM.pdf]

Additional file 13: Fig. S9

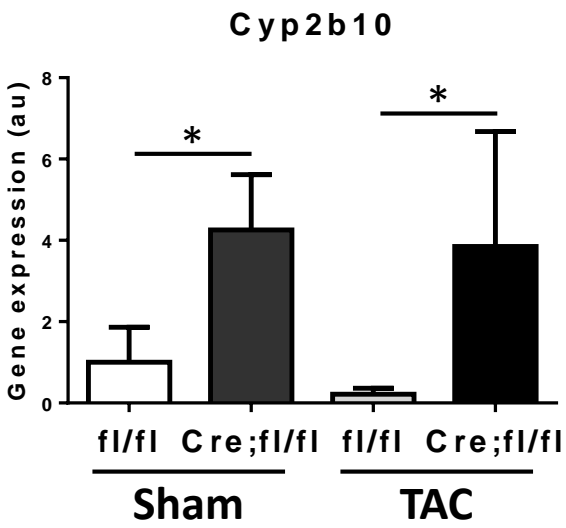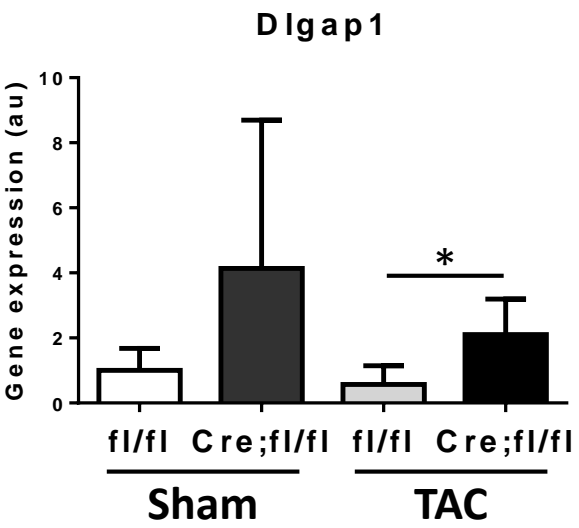

Additional file 14: Fig. S10

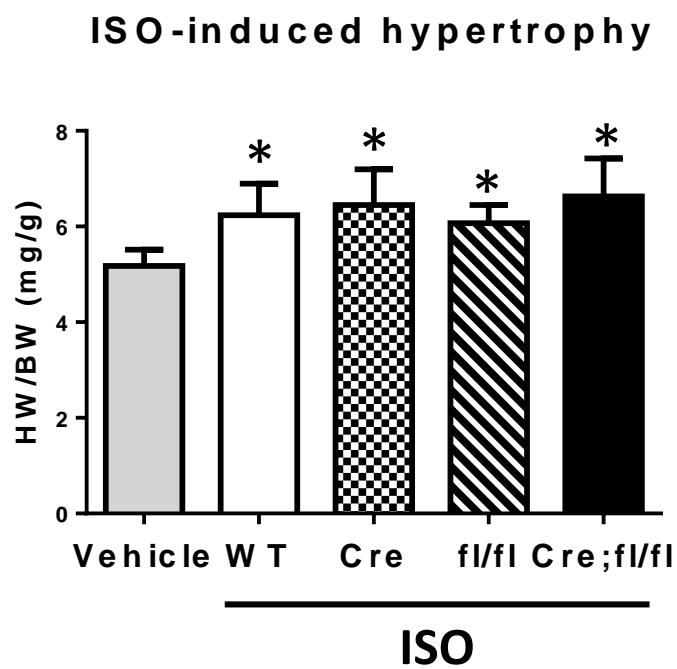

Supplement: Supplementary file 14 — Additional file 14: Fig. S10. Effect of HP1γ KO on ISO-induced heart growth. ISO (5 μg/g) was injected subcutaneously once a day for 6 days and hearts were harvested. Same volume of saline was injected as a vehicle control. Since there was no difference of HW normalized by BW at base line, all genotype of animals with vehicle injection are grouped as a vehicle control group. Upon ISO treatment, all genotype showed a significant increase in HW/BW compared to vehicle control; however, there was no difference between genotype, indicating that HP1γ KO doesn’t have a significant effect on ISO-induced heart growth. * p < 0.05 vs vehicle control. [file 13072_2018_187_MOESM14_ESM.pdf]
