# Supplementary material for: Deletion of HP1γ in cardiac myocytes affects H4K20me3 levels but does not impact cardiac growth
Source: Epigenetics Chromatin. 2018 Apr 17;11:18. doi: 10.1186/s13072-018-0187-z (PMC5905015; doi:10.1186/s13072-018-0187-z)
Supplement: Supplementary file 8 — Additional file 8: Table S1. Comparison of cardiac function in HP1γ KO mouse. Echo cardiograms were taken at 8 weeks. All data are presented as mean ± SD. No significant difference was detected on parameters we measured by one-way ANOVA followed by multiple comparison. [file 13072_2018_187_MOESM8_ESM.pdf]

Additional file 7: Table S1

	WT	Cre	fl/fl	Cre;fl/fl
Heart Rate (bpm)	433 ± 20	458 ± 12	436 ± 50	422 ± 42
End-Systolic Diameter (mm)	3.0 ± 0.25	2.6 ± 0.12	2.6 ± 0.38	2.6 ± 0.52
End-diastolic Diameter (mm)	3.9 ± 0.39	3.8 ± 0.28	3.7 ± 0.32	3.7 ± 0.35
Stroke Vol. (ul)	39.6 ± 6.8	37.7 ± 8.3	33.8 ± 7.0	33.6 ± 8.2
Ejection Fraction (%)	52.5 ± 3.9	58.5 ± 7.1	56.5 ± 7.5	58.1 ± 12
Fraction Shortening (%)	26.7 ± 2.5	30.8 ± 4.9	29.6 ± 5.3	30.9 ± 7.9
Cardiac Output (ml/min)	16.9 ± 2.2	17.4 ± 4.1	15.0 ± 4.0	14.0 ± 3.0
Left Ventricular Mass (mg)	129 ± 24	119 ± 21	102 ± 19	96.6 ± 29
